# Single residue modulators of amyloid formation in the N-terminal P1-region of α-synuclein

**DOI:** 10.1038/s41467-022-32687-1

**Published:** 2022-08-25

**Authors:** Sabine M. Ulamec, Roberto Maya-Martinez, Emily J. Byrd, Katherine M. Dewison, Yong Xu, Leon F. Willis, Frank Sobott, George R. Heath, Patricija van Oosten Hawle, Vladimir L. Buchman, Sheena E. Radford, David J. Brockwell

**Affiliations:** 1 Astbury Centre for Structural Molecular Biology, School of Molecular and Cellular Biology, Faculty of Biological Sciences, University of Leeds, Leeds, LS2 9JT UK; 2 Astbury Centre for Structural Molecular Biology, School of Physics & Astronomy, University of Leeds, Leeds, LS2 9JT UK; 3 School of Biosciences, Cardiff University, Cardiff, CF10 3AX UK; 4 Belgorod State National Research University, 85 Pobedy Street, Belgorod, 308015 Belgorod Region Russian Federation

**Keywords:** Protein aggregation, Intrinsically disordered proteins, Structural biology, Parkinson's disease

## Abstract

Alpha-synuclein (αSyn) is a protein involved in neurodegenerative disorders including Parkinson’s disease. Amyloid formation of αSyn can be modulated by the ‘P1 region’ (residues 36-42). Here, mutational studies of P1 reveal that Y39A and S42A extend the lag-phase of αSyn amyloid formation in vitro and rescue amyloid-associated cytotoxicity in *C. elegans*. Additionally, L38I αSyn forms amyloid fibrils more rapidly than WT, L38A has no effect, but L38M does not form amyloid fibrils in vitro and protects from proteotoxicity. Swapping the sequence of the two residues that differ in the P1 region of the paralogue γSyn to those of αSyn did not enhance fibril formation for γSyn. Peptide binding experiments using NMR showed that P1 synergises with residues in the NAC and C-terminal regions to initiate aggregation. The remarkable specificity of the interactions that control αSyn amyloid formation, identifies this region as a potential target for therapeutics, despite their weak and transient nature.

## Abstract (web summary)

The authors of this work characterize the effect of amino acid substitution on α-synuclein (α-Syn) aggregation. Residues 38 and 42 (in addition to 39) within the P1 region of α-Syn affect amyloid formation. The effect of substitution at position 38 is dependent on the amino-acid introduced, suggesting that specific interactions control α -Syn aggregation.

## Introduction

Alpha synuclein (αSyn) is a 140 amino acid intrinsically disordered protein (IDP) associated with neurodegenerative diseases that include Parkinson’s disease (PD), Multiple System Atrophy and Dementia with Lewy bodies^1^. These diseases affect more than 1% of the world’s population above 60 years of age, and PD is currently the second most common neurodegenerative disorder after Alzheimer’s disease^2^. Many studies have shown that the pathology of PD is associated with the presence of cytotoxic αSyn oligomers^3^, combined with the generation of amyloid fibrils^4,5^ and the formation of αSyn-containing Lewy bodies in the *substantia nigra* of PD patients^6,7^. The first synuclein protein was discovered more than 30 years ago and a specific genetic association of αSyn and PD was reported in 1997^8^. Since this time, αSyn has been studied intensively in vitro^9,10^, in silico^11^, in cells^12^ and in animal models^13^, yet the molecular mechanism(s) of αSyn aggregation that lead(s) to its involvement in different disorders remains unclear.

**Fig. 1:**
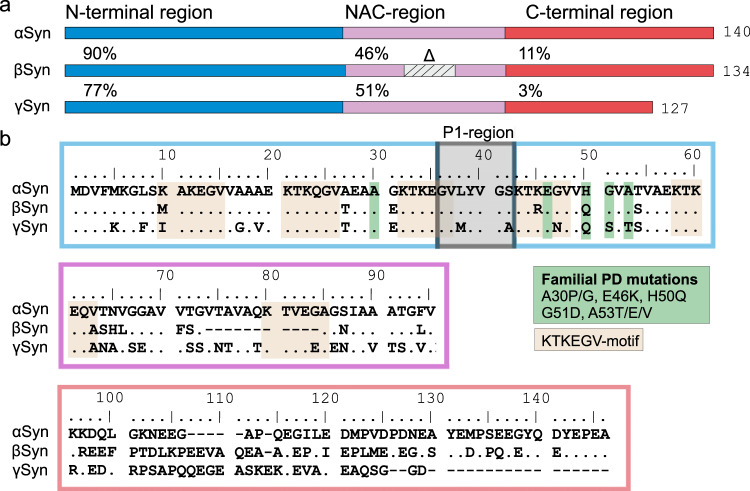
Sequence alignment of α-, β- and γSyn. **a** Each protein comprises three regions: the amphipathic N-region (blue), the amyloidogenic NAC region (pink) and the acidic C-terminal region (red). The sequence identity of βSyn and γSyn to αSyn for each region is shown. The hatched pink region in the centre of NAC for βSyn (Δ) depicts residues in the highly aggregation NAC core (residues 74–84) that are deleted in this sequence. The number of residues in each protein is shown on the right-hand side. **b** Sequence alignment for each of the paralogues (outlines coloured according to **a**. Positions of common familial PD mutations of αSyn are highlighted in green and each of the KTKEGV motifs^15^ involved in membrane binding is highlighted in brown. The ‘master controller’ P1 region is also highlighted in a grey shaded box. “–“ represents a deletion of a residue and “.” represents residue identity at that site.

The primary sequence of αSyn can be divided into three distinct regions: a basic N-terminal region (residues 1–60), which is involved in membrane binding and contains five (of a total of six) copies of an 11-residue motif (xxKTKEGVxxx)^14,15^; the non-amyloid β component (NAC) (residues 61–95), which is highly aggregation prone^16^ and the acidic C-terminal region (residues 96–140) involved in binding Ca^2+^ and other metal ions^17^ (Fig. 1a, b). The NAC region is critical for amyloid formation, consistent with the high hydrophobicity and aggregation propensity of its sequence^18–20^. NAC forms the core in all αSyn fibrils whose structure has been determined to date using solid-state NMR or cryo-EM^5,21^. Despite the long-known importance of NAC in aggregation, it is becoming increasingly apparent that residues/regions which flank NAC, including some (but not all) of the eight-known common familial PD mutations, can affect its rate of aggregation^22–26^. Recently, we described the critical role of a 7-residue sequence (P1: residues 36–42 (^36^GVLYVGS^42^)) (Fig. 1b) in the N-terminal region of αSyn that is required for amyloid formation in vitro at neutral pH on our experimental timescale and whose deletion is protective in a *C. elegans* model of PD^27^. An additional 13-residue sequence C-terminal to P1 (named P2; residues 45–57^27^), also known as the “pre-NAC” region (residues 47–56^28^), was also shown to play a role in controlling amyloid formation, such that deletion of both P1 and P2 results in an αSyn sequence that loses its ability to aggregate into amyloid at both acidic (pH 4.5) and neutral pH over a timescale of at least 110 h, while deletion of P2 alone does not significantly affect αSyn aggregation at either pH^27^. A peptide that includes both P1 and P2 (residue 36–55) has been shown in vitro^29^ and in silico^30^ to form β-hairpin structures that self-assemble into oligomers^29^. The binding of a β-wrapin protein to αSyn in this region resulted in β-hairpin formation in the bound state of αSyn which arrests fibril formation in vitro and prevents αSyn-associated cytotoxicity in *Drosophila* and primary cortical neurones^31,32^. The P1 region in monomeric αSyn is also involved in binding various chaperones (centred on residue Y39) that prevent fibril formation^33^, and binding of chaperones to P1 can result in fibril disaggregation^34^. Finally, P1 contains a SUMO interaction motif, and binding of SUMO to P1 prevents fibril formation, in vitro, in cells and in flies^35^. Together these results confirm the role of the P1 region in controlling αSyn amyloid formation in vitro, as well as in a biological setting.

αSyn has two paralogues, βSyn and γSyn, with a high sequence identity in the N-terminal region (90% and 77% homology to αSyn, respectively)^36^ (Fig. 1a). Compared to αSyn, these proteins have a reduced propensity to form amyloid, showing no fibril formation at neutral pH^18^, and the presence of either βSyn and γSyn (at 4:1 molar excess) inhibits αSyn fibrillation^18,37^. This feat may be rationalised for βSyn by the deletion of 11 residues within the NAC region (Fig. 1b)^38^. By contrast, γSyn shows no obvious differences compared with αSyn in the P1 region that explains its low amyloid propensity (Fig. 1b), and it retains a NAC region predicted to have a high aggregation propensity, especially in the central-NAC core region (residues 65–79) (Fig. 1a, b)^20,25,28^. Finally, although γSyn has a truncated C-terminal region, the fibrillation of αSyn is accelerated upon deletion of 10–45 C-terminal residues^39^, demonstrating that this sequence alteration alone cannot rationalise its low amyloid propensity.

Here, to understand the role of individual amino acids within P1 in controlling the amyloid propensity of αSyn, we performed an alanine scan of the seven residue P1 sequence. P1 was chosen for our analysis (over P2) given the evidence described above that P1 plays a major role in driving aggregation into amyloid, whilst the adjacent P2 region acts only in synergy with P1^27^. For each variant, we measured the in vitro aggregation rate into amyloid and seeding capacity (using Thioflavin T (ThT) fluorescence assays) at pH 7.5, high molecular weight aggregate yield using a pelleting assay, aggregate morphology (using transmission electron microscopy (TEM) and atomic force microscopy (AFM)), and the effect of aggregation on a phenotypic trait in a *C. elegans* model of PD^40^. The results revealed a remarkable sensitivity of amyloid formation kinetics on the sequence of P1, with residue S42 (along with the previously identified Y39^41^) individually able to significantly extend the lag-phase of fibril assembly when substituted with Ala. In addition, we show that the identity of residue 38 tunes the rate of amyloid formation, with L38I forming fibrils more rapidly than WT αSyn, L38A having no effect, and L38M (the equivalent residue found in γSyn) significantly retarding aggregation into amyloid such that long and straight fibrils characteristic of amyloid are not detected under the conditions explored. The converse experiment, in which residues in the P1 region of γSyn were switched to their equivalents in αSyn (M38L, A42S alone and M38L/A42S together) did not enable amyloid formation, under the conditions used, highlighting a complex interplay of compensatory interactions that define the amyloid propensity of the protein. Using peptide binding experiments, NMR PREs (Paramagnetic Resonance Enhancement) and analysis of chemical shift perturbations (CSPs), we show that P1 synergises with residues in the NAC and C-terminal region to create conformers capable of initiating amyloid formation. Together the results demonstrate that the early intra- and inter-molecular interactions that control amyloid formation kinetics of αSyn are remarkably specific, despite their weak and transient nature. Such interactions, at least for the P1 region, depend crucially both on the location of the amino acid within the sequence of P1 and the identity of the sidechain at position 38.

## Results

### The sequence of P1 drives amyloid formation

**Fig. 2:**
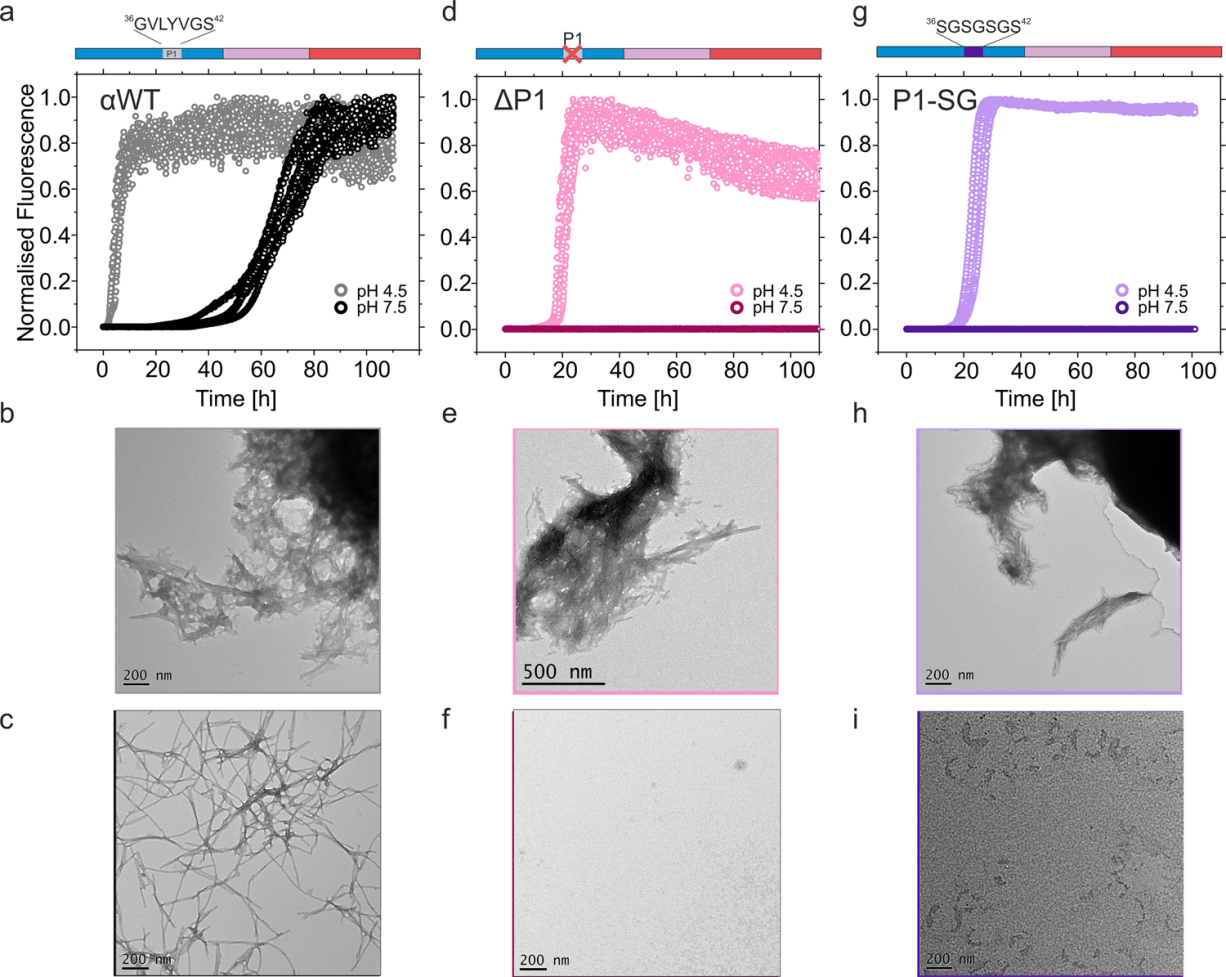
The sequence of P1 (^36^GVLYVGS^42^) is crucial for amyloid formation. Fibrillation of WT αSyn at pH 7.5 or 4.5 measured by **a** ThT fluorescence and **b**, **c**, negative stain TEM, showing the end points of the experiment at **b** pH 4.5 (20 mM sodium acetate) and **c** pH 7.5 (20 mM Tris-HCl) (each in 200 mM NaCl). **d**–**f**, as for **a**–**c**, but for ΔP1. **g**–**i**, as for **a**–**c**, but for P1-SG-αSyn. Each condition was measured in at least triplicate. Note that short, clumped fibrils result at pH 4.5, presumably as fibril formation is rapid and the pH is close to the pI of the proteins. The results show that the sidechains of P1 are essential for rapid fibril formation at both pH values. A schematic of the sequence of αSyn is shown above each ThT plot, with the N-terminal region in blue, NAC in pink and the C-terminal region in red. The presence or absence of the P1 region is highlighted in each case. % pellet and t_50_ values for these experiments are shown in Supplementary Table 1a. Source data are provided as a Source Data file.

In previous work, we showed that deletion of the seven-residue P1 sequence ^36^GVLYVGS^42^ in the N-terminal region of αSyn (in the variant, ΔP1) prevents the protein from forming amyloid fibrils at neutral pH with little, or no, fibrils being observed after at least 100 h incubation (at pH 7.5, 200 mM NaCl), and significantly retards amyloid fibril formation at lower pH (pH 4.5, 200 mM NaCl)^27^ (Fig. 2a–f and Supplementary Table 1a). Amyloid formation is driven by the high thermodynamic stability of the cross-β amyloid core, yet is under kinetic control and so we emphasise here that the (in)ability of various sequences to form amyloid fibrils in this study reveals the amyloid propensity of each sequence relative to each other and to wild-type αSyn under our experimental conditions (100 μM αSyn at 37 °C, 20 mM Tris-HCl, 200 mM NaCl, pH 7.5, 600 rpm and 110 h incubation time unless otherwise stated). Deleting P1 both removes seven residues (which may make specific interactions with other regions) and alters the spacing of the KTKEGV motifs in the N-terminal region. To differentiate these effects, the P1 sequence was replaced with the seven-residue sequence ^36^SGSGSGS^42^ (creating the protein, P1-SG-αSyn in which P1 is replaced with a dynamically disordered, soluble linker lacking secondary structure) and the amyloid formation kinetics of this variant were measured using ThT fluorescence. At pH 7.5, these experiments revealed that P1-SG-αSyn behaved similarly to ΔP1, with no detectable increase in ThT fluorescence observed over 100 h (Fig. 2g–i). The lack of positive ThT signal, could be due to the inability of fibrils of this variant to bind to the extrinsic fluorophore or due to changes in the fluorophore’s photophysical properties when bound, yielding a false negative reading. To obviate this possibility we used a series of orthogonal and complementary assays (ThT, TEM and AFM imaging and a pelleting assay) to detect and characterise high molecular weight material^42,43^. The rationale of using these assays is described below (Methods). At pH 4.5 slower kinetics were observed for P1-SG compared to αSyn WT, yielding short and clumped fibrils (visualised using negative stain TEM) (Fig. 2g–i). The percentage aggregated material determined by pelleting assays (see Methods) are listed in Supplementary Table 1a. These experiments indicate that the effect of P1 in driving amyloid formation of αSyn is sequence-specific. This is consistent with previous results which showed that replacing both the P1 and P2 regions with a GS linker (in the construct ΔΔ-SG) also ablates detectable fibril formation over this timescale at both pH values, and that deleting a 7-residue sequence elsewhere in the N-terminal region (named ΔC1 (residues 14-20) has no effect on fibrillation kinetics^27^. Hence, the amino acids that comprise the P1 sequence must play a vital role in controlling the amyloid propensity of this 140 residue IDP.

**Fig. 3:**
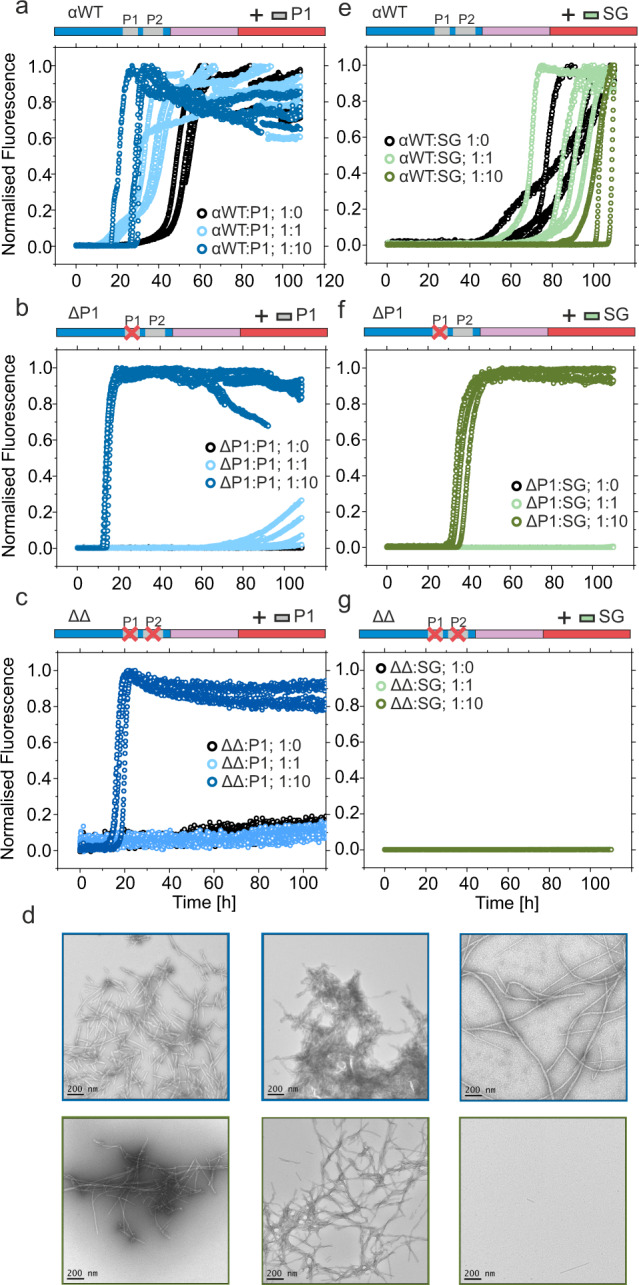
Amyloid formation kinetics of WT αSyn, ΔP1 and ΔΔin the presence of the peptides P1 or P1-SG. Amyloid formation kinetics of **a**, **e** WT αSyn, **b**, **f** ΔP1 and **c**, **g** ΔΔ in the presence of different concentrations of P1-peptide (**a**–**c**) or peptide P1-GS (**e**–**g**). All experiments were carried out using 100 μM αSyn and peptide concentrations of 0 μM, 100 μM or 1 mM, at pH 7.5, 200 mM NaCl, 37 °C, 600 rpm. Note that under conditions of no or low amyloid formation, data points for different conditions overlay. Representative negative stain TEM images of samples with 10-fold molar excess of peptide taken at the end point (110 h) of one biological replicate (*n* = 2) are shown (**d**). Top, with peptide P1; lower, with peptide P1-SG. % pellet and t_50_ values are shown in Supplementary Table 1b. Source data are provided as a Source Data file.

To investigate in more detail how P1 exerts its effects on αSyn amyloid formation, a synthetic peptide with the sequence of P1 (with four residue extensions taken from the natural sequence of αSyn added to the N- and C-termini to enhance its solubility (Ac-KTKE-GVLYVGS-KTKE-NH_2_, named P1-peptide) was added to WT αSyn, and the αSyn variants ΔP1, and ΔΔ (deletion of both P1 and P2 in αSyn). The effect of the addition of the P1 sequence in trans (i.e. P1-peptide) on the aggregation kinetics of each protein was determined. Interestingly, the P1-peptide increased marginally the rate of fibril formation of WT αSyn at pH 7.5 when added in equimolar or 10-fold molar excess (*t*_50_ of 51.3 ± 2.2; 40.1 ± 7.2 and 26.6 ± 3.9 h with 0, 1:1 or 1:10 (mol:mol) αSyn:P1-peptide) (Fig. 3a, Supplementary Table 1b). The peptide had an even greater effect on ΔP1, stimulating aggregation to commence within 110 h when added in an equimolar ratio and resulting in rapid fibril formation (*t*_50_ of 14.4 ± 0.7 h) when added in 10-fold molar excess (Fig. 3b, Supplementary Table 1b). For ΔΔ, the addition of the P1-peptide in trans was also able to induce fibril formation, although only when added in 10-fold excess (Fig. 3c, Supplementary Table 1b). TEM images confirmed that fibrils were formed from all three proteins in the presence of 10-fold excess of the peptide (Fig. 3d, top row). Control experiments showed that P1-peptide alone does not self-assemble into high-order aggregates over the timescale of the experiment, as judged using far UV CD, ThT fluorescence and negative stain TEM (Supplementary Fig. 1a,b). Together, these data indicate that the P1-peptide is able to enhance αSyn amyloid formation kinetics by interacting with one or more regions of the protein, replacing the effect of the P1 sequence on intra-/inter-molecular interactions in trans. Control experiments performed by adding the peptide P1-SG (which also does not aggregate in isolation (Supplementary Fig. 1c, d)), with the sequence Ac-KTKE-SGSGSGS-KTKE-NH_2_, was much less efficient than the P1-peptide in inducing aggregation of ΔP1 (*t*_50_ of 37.0 ± 2.5 and 14.4 ± 0.7 h, respectively), and had no effect on the ability of WT αSyn and ΔΔ to form amyloid (Fig. 3d–g, Supplementary Table 1b). This adds further weight to the importance of the specific sequence of P1 in driving amyloid formation, with the rate of amyloid formation depending both on the sequence of the peptide and the protein to which it was added.

### Determining the binding mode between P1-peptide and the proteins αSyn and ΔP1

**Fig. 4:**
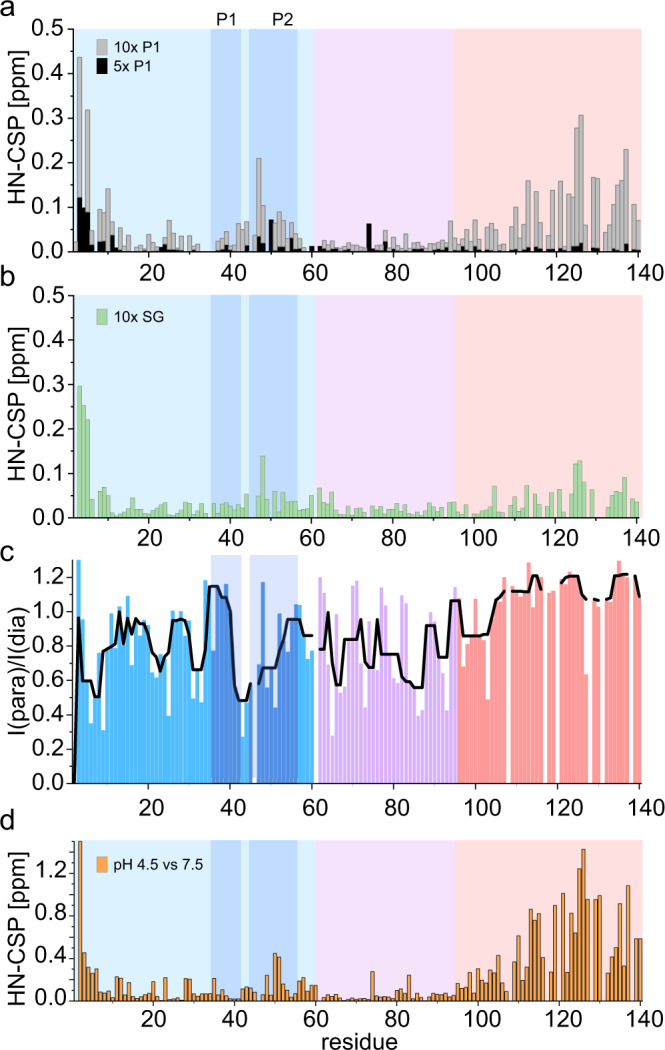
The binding of peptide P1 alters the conformational ensemble of WT αSyn. HN-CSPs of WT αSyn upon the addition of **a** a 5- or 10-fold molar excess of peptide-P1, **b** a 10-fold molar excess of peptide P1-SG and **c** NMR PREs of ^15^N-αSyn upon the addition of equimolar MTSL-labelled peptide-P1. The black line represents the median value over a rolling window of five residues. **d** Difference in chemical shifts of WT αSyn at pH 7.5 and 4.5. All experiments were performed in 20 mM Tris-HCl buffer, pH 7.5, 200 mM NaCl, 15 °C. The N-terminal region is shaded light blue, NAC is in purple, and the C-terminal region is in red. The P1 and P2 regions are shown in darker blue. Source data are provided as a Source Data file.

To identify the region(s) of WT αSyn and ΔP1 that interact with the P1-peptide, and to determine how peptide binding may increase the rate of amyloid formation, ^1^H-^15^N-HSQC NMR experiments were performed in which P1-peptide (non-labelled) was mixed with ^15^N-labelled αSyn and the interaction interface(s) between the peptide and protein was measured using HN-chemical shift perturbation (HN-CSP). In the presence of a 10-fold molar excess of the P1-peptide significant HN-CSPs are observed for WT αSyn resonances corresponding to the N-terminal ∼15 residues, the P2 region (residues 45–57) and the C-terminal region (residues ∼100–140) of ^15^N-αSyn (Fig. 4a), with P1 binding resulting in the largest CSPs for the N-terminal ∼10 residues. These changes (which are dependent on the concentration (Fig. 4a) and sequence of the peptide (Fig. 4b)) could result from direct binding of the peptide to the protein at these sites, or from indirect effects such as conformational changes in regions distant from the binding site. These scenarios can be distinguished using NMR PRE experiments, which are ideal for detecting transient interactions between a spin-labelled ligand and nearby atoms in a binding partner, with a distance cut-off of 25 Å on Cα^44^. Accordingly, when ^14^N P1-peptide N-terminally labelled with S-(1-oxyl-2,2,5,5-tetramethyl-2,5-dihydro-1H-pyrrol-3-yl)methyl methanesulfonothioate (MTSL) was mixed with ^15^N-labelled WT αSyn clear evidence for enhanced relaxation of residues in the N-terminal ~100 residues (including the N-terminal and NAC regions) of αSyn was obtained (Fig. 4c), suggestive of multiple binding sites for the P1-peptide in these regions. Native nESI-MS experiments showed 1:1 binding between protein and peptide (mass of the complex 16,167.54 ± 0.17 Da; theoretical mass 16,168 Da), but no higher order binding processes (peptide is in 10 times molar excess in solution, Supplementary Fig. 2a). The nESI-MS, HN-CSP and PRE data together suggest a binding mode in which one peptide molecule binds to different locations within the N-terminal and NAC regions of αSyn (resulting in CSPs of different magnitude which, without further investigation, cannot be interpreted here). Notably, a strong PRE effect was not observed for residues in the acidic C-terminal region, despite the preponderance of positively charged residues in the natural KTKE sequences that flank the P1 sequence used here to enhance the solubility of the peptide (Fig. 4c). Consequently, the observed HN-CSPs in the C-terminal region of WT αSyn observed upon P1 binding must result from changes in long-range intra-molecular interactions of the N-terminal and/or NAC regions with the C-terminal region upon peptide binding (note that inter-molecular interactions between αSyn molecules are not observed using NMR PREs under the conditions and αSyn concentrations employed here^27^). Such a scenario is consistent with previous results that have shown interactions between the N-terminal, NAC and C-terminal regions using intra-molecular NMR PRE measurements^27,39,45–47^. Importantly, the HN-CSPs observed upon binding the P1 peptide to WT αSyn show a striking similarity to those observed when the pH is decreased from pH 7.5 to 4.5 (Fig. 4d) which also enhances the rate of amyloid formation (Fig. 2a and ref. 10). Further analysis of the HN-CSPs of individual residues (Supplementary Fig. 3) showed that most cross-peaks move on the same vector when comparing the CSPs with pH change or upon P1 addition. However, there are exceptions in which no HN-CSPs are observed for the addition of the peptide, but are observed upon changes in pH (e.g. Q122), or where chemical shifts move in opposite directions (e.g. D135) suggesting similar, but not identical, conformational changes.

Addition of 10x P1-peptide to ΔP1 results in an even faster acceleration of amyloid formation compared with the effects observed with WT αSyn (Fig. 3b). We therefore also investigated the binding mode between these two molecules using NMR and native nESI-MS. HN-CSP analysis (Supplemental Fig. 4a) showed a similar pattern of chemical shifts (with significant chemical shifts observed for the N-terminal ~15 residues, the P2 region and C-terminal region), but with a marked change in the relative amplitudes of the CSPs in the P2 (increased) and C-terminal (decreased) regions. Most notably, an increased magnitude of CSPs is observed for the P2 region (residues 45–57) compared with those obtained with WT αSyn, consistent with an interaction between the P2 region and the P1 peptide, potentially by the formation of a β-hairpin in this region^29,30^. The binding mode investigated by NMR PREs and native nESI-MS also indicates a diffuse binding of the P1 peptide throughout the N-terminal and NAC region (~100 residues) with a 1:1 binding as described for WT αSyn (observed mass for the complex of 15,492.23 ± 0.25 Da; theoretical mass 15,492 Da) (Supplementary Fig. 4b,c and Supplementary Fig. 2a). Collision-induced dissociation (CID) experiments indicated a slightly tighter binding for P1 peptide and ΔP1 compared with αSyn WT, with CID_50_ values of 40 V and 44 V for αSyn WT and ΔP1, respectively for protein-peptide dissociation (Supplementary Fig. 2b–d). Together, these results suggest that the P1-peptide accelerates the aggregation of ΔP1, and enhances the amyloid propensity of WT αSyn, by competing with the long-range intra- and/or inter-molecular interactions between the N- and C-terminal regions of αSyn that protect the protein from amyloid formation^39,45^. Differences are observed in the HN-CSPs for αSyn WT and ΔP1 and further work is required to understand whether distinct molecular mechanisms underlie this observation. Nonetheless, our data demonstrate that the P1 sequence can enhance amyloid formation for these variants when added in trans.

### Identifying key residues within the P1-region that control amyloid formation

While the above data confirm that the seven residue P1 sequence plays a key role in modulating the rate of αSyn fibril formation, the relative importance of each of the residues in this sequence remained unresolved. Previous experiments have shown that single residue substitutions in different regions of αSyn can have significant effects on the rates of amyloid formation in vitro and in vivo, as exemplified by the eight familial PD point mutations which induce early onset disease, and post-translational modifications in the N-terminal, NAC and C-terminal regions which also change the rate of amyloid formation^48–51^. Deep mutational scanning has also highlighted the role of the ~90 N-terminal residues in αSyn aggregation in yeast^52^, with single residue changes also affecting membrane binding in vitro^49^. Tyr39, which lies in the centre of the P1 region (Fig. 1b), has been shown previously to play an important role in αSyn amyloid formation and toxicity. For example, Y39A αSyn forms amyloid more slowly than WT αSyn (in 100 mM sodium phosphate buffer, 100 mM NaCl, pH 7.4)^41^, and αSyn phosphorylated at Y39 is enriched in brain tissues and Lewy bodies in the *substantia nigra* and *striatum* of PD patients^53^. In addition, Y39 forms the epicentre for binding of chaperones (SecB, Skp) that protect αSyn from aggregation^33^ and this same region is involved in binding Hsp70 for fibril disassembly^34^. Finally, distinct fibril structures were observed using cryoEM for αSyn fibrils formed in vitro from protein that is phosphorylated at Y39 compared with its WT counterpart^21,54^.

**Fig. 5:**
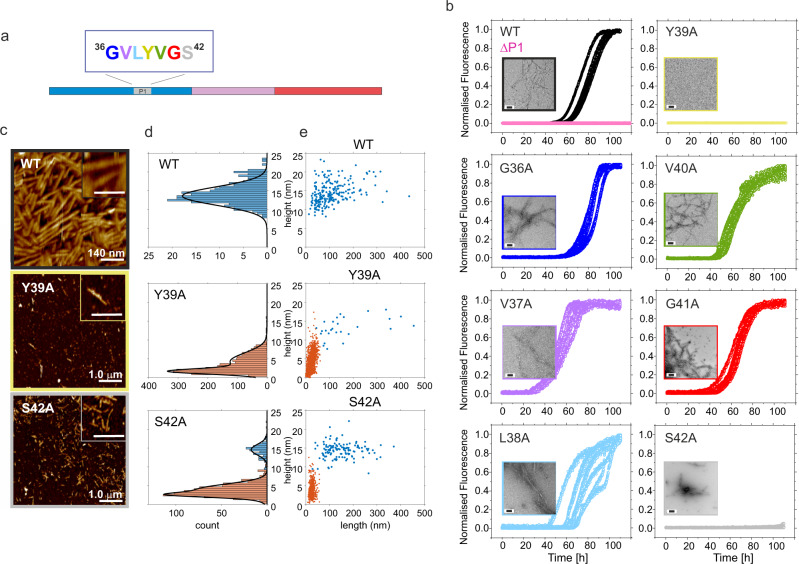
Alanine scan to identify residues in P1 region important for amyloid formation. **a** Schematic showing the sequence of the P1 region of αSyn. **b** Amyloid kinetics of the seven Ala variants in the P1 region of αSyn determined using 100 μM αSyn at 37 °C, 20 mM Tris-HCl, 200 mM NaCl, pH 7.5, 600 rpm, coloured as in **a**. Data for WT αSyn and ΔP1 are shown for comparison. Inserted are representative TEM images taken at the end point of one biological replicate (*n* = 2). Scale bar = 200 nm. Further images for Y39A and S42A are shown in Supplemental Fig. 5. **c** AFM images of WT αSyn (top), Y39A (middle) and S42A (bottom) at the endpoint (110 h) of the fibril growth experiment. The inset shows an expanded scale (scale bar = 50 nm). **d** Height and **e** length/height distributions of the AFM samples (WT = 232; Y39A = 2355; S42A = 898 counts). Fibrillar species are illustrated in blue, monomers and oligomers are coloured in orange. Note that monomers/oligomers were not detected for WT αSyn. % pellet and t_50_ values for the aggregation assays are shown in Supplementary Table 1c. Source data are provided as a Source Data file.

To compare the role of Y39 alongside each of six other residues of P1 (Fig. 5a) in αSyn fibril assembly kinetics, each residue in P1 was substituted individually with alanine and the rate of amyloid formation of each variant was assessed using ThT fluorescence. The results revealed that five of the seven alanine substitutions had little/no effect, forming fibrils with similar (G36A, L38A), or slightly faster (V37A, V40A, and G41A) rates than WT αSyn (Fig. 5b and Supplementary Table 1c). As expected^41,55^ Y39A did not form amyloid fibrils and, remarkably, S42A also abolished detectable ThT fluorescence over the duration of the experiment (110 h) (Fig. 5b, yellow and grey, respectively). These observations were verified by quantifying the yield of pelletable material at the end of the experiment (Supplementary Table 1c) and by imaging samples at the end point of these experiments by TEM (Fig. 5b, inserts) and AFM (Fig. 5c). The end point morphology of the aggregated species of these variants was distinct from those of wild-type. WT αSyn formed fibrils with a height (measured using AFM (Methods)) of 13.6 ± 3.4 nm (Supplementary Table 3). Y39A yielded spherical particles capturing broad height distributions centred on 1.6 ± 1.0 nm and 4.3 ± 3.3 nm consistent with monomers and oligomers with a small fraction (1.1%) of short fibrils with height 13.5 ± 3.8 nm (Fig. 5c–e, middle; Supplementary Table 3). S42A also yielded spherical particles distributed at 2.5 ± 0.8 nm and 3.9 ± 2.1 nm, along with a minor population (~15%) of short fibrils similar in height to those formed from WT αSyn (heights of 14.6 ± 2.0 nm and lengths 153 ± 74 nm) (Fig. 5c–e, lower; Supplementary Table 3). Finally, the % pelletable material (25% and 10% for Y39A and S42A, respectively) indicate the additional formation of amorphous aggregates at the end of the experiment for these variants as seen by TEM (Fig. 5b and Supplementary Fig. 5). Together, the results show that single residue substitutions with Ala at just two sites within the P1 region are able to reduce the ability of αSyn to assemble into amyloid-like fibrils in vitro at neutral pH, at least on the timescale used here, demonstrating a key role of these specific sidechains in one or more stages of the assembly reaction. Notably, the pelleting assay used here does not resolve low and high molecular weight oligomers from monomer and dimers^43,56^. Further studies will be required to characterise the effects that these substitutions have on oligomer formation in the early stages of the amyloid cascade^42^.

Amyloid formation of αSyn and other proteins is typically described by a sigmoidal growth curve defined by a lag-phase in which nuclei and oligomers form; an elongation phase, dominated by fibril growth and secondary nucleation; and a plateau/stationary phase in which mature fibrils are in equilibrium with soluble monomer/oligomers^57^. Adding pre-formed seeds obviates the need for nucleation and removes the rate-limiting nucleation phase if the monomer is seed-elongation-competent. To determine which stage(s) of αSyn assembly are blocked by the variants created here, the aggregation kinetics of ΔP1, P1-SG-αSyn and the seven alanine variants (G36A, V37A, L38A, Y39A, V40A, G41A, S42A) were each measured in the presence of 10% (*w/w*) fibril seeds formed from WT αSyn (Methods). The results showed that all of the alanine variants are able to elongate WT αSyn seeds, resulting in similar rates of elongation (Supplementary Fig. 6a–i, Supplementary Table 2a), including Y39A and S42A which did not form detectable amyloid fibrils in 110 h without seeds (compare Fig. 5b yellow, grey with Supplementary Fig. 6c, i). Aggregation of Y39A and S42A is thus blocked at an early stage in fibril formation, e.g., at the nucleation and/or oligomerisation level, consistent with the accumulation of oligomers for these proteins observed using AFM (Fig. 5c). In contrast, ΔP1 and P1-SG-αSyn did not result in successful cross-seeding with WT αSyn pre-formed fibrils (Supplementary Fig. 6j, k, Supplementary Table 2b), indicating that these variants are not able to nucleate fibril growth, nor are they able to elongate WT αSyn fibrils, presumably because their sequence is incompatible with the architecture of WT αSyn fibrils. Given that P1 is intimately or peripherally involved in the cores of all WT αSyn fibril structures determined to date, including fibrils formed in vitro^21^ and purified from patients with multiple system atrophy^5^, the results highlight the importance of the P1 sequence both in the nucleation of fibril growth and in stabilising the cross-β structure of the amyloid fibrils that form.

### Comparison of the P1 regions of αSyn and γSyn reveals residue 38 as an additional tuner of amyloid formation kinetics

**Fig. 6:**
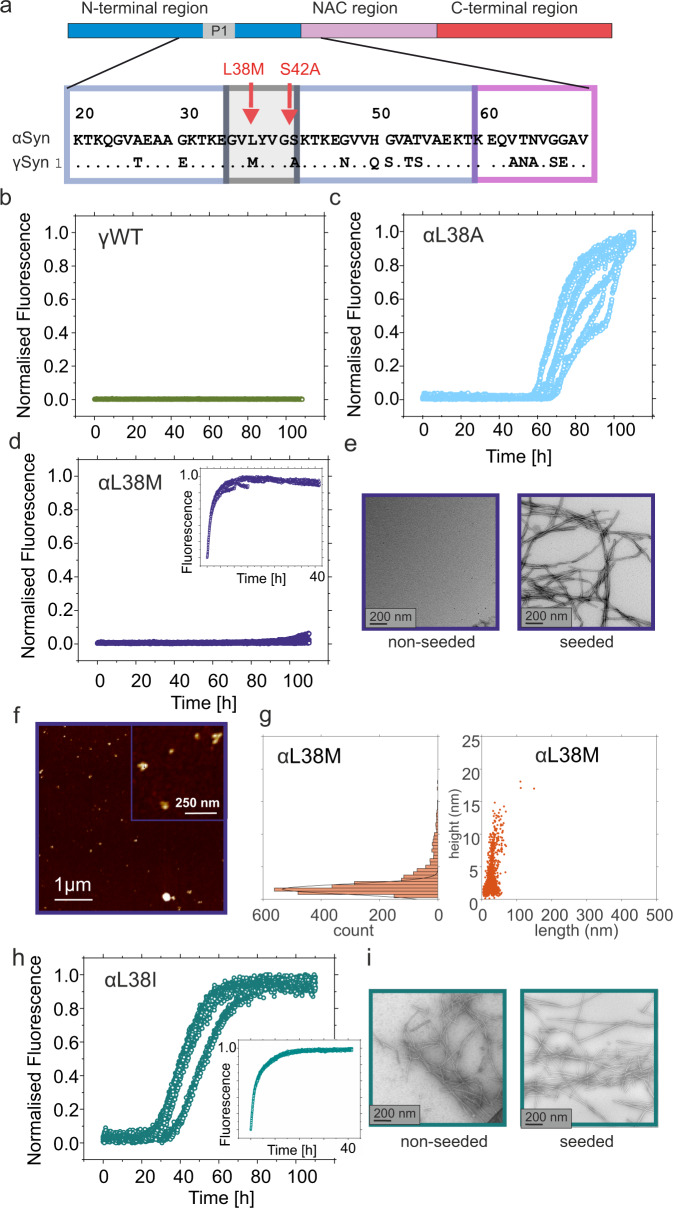
αSyn aggregation rate is dependent on the identity of residue 38. **a** Sequence alignment showing that the P1 regions of αSyn and γSyn differ at two positions, residues 38 and 42. Aggregation kinetics of **b** γSyn, **c** αSyn L38A (reproduced for clarity from Fig. 5b), and **d** αSyn L38M. The inset shows seeding of L38M with 10% (*w/w*) preformed WT αSyn seeds. **e** TEM images of αSyn L38M incubated in the absence (left hand side) or presence (right hand side) of 10% (*w/w*) preformed seeds of WT αSyn. **f**, **g** AFM image and height/length distribution analysis (*n* = 2167 objects analysed in one image) of the products of incubation of L38M (without seeds). Amyloid formation kinetics (**h**) and TEM images (**i** left hand side) for αSyn L38I incubated without seeds. The inset shows seeding of L38I with 10 % (*w/w*) preformed WT αSyn seeds, with the TEM image alongside (**i** right hand side). TEM images of each sample taken at the end of the reaction (110 h) of one biological replicate (*n* = 2) All reactions were carried out at pH 7.5, 200 mM NaCl, 37 °C, shaking (600 rpm) (de novo growth) or quiescent (seeded growth) each using 100 μM αSyn. See also Supplementary Fig. 8. % pellet and t_50_ values are shown in Supplementary Table 1d. Source data are provided as a Source Data file.

The data presented above show that individual residues within P1 can modulate the rate of aggregation of αSyn into amyloid fibrils with the rate of fibrillation dependent on the identity of the sidechain at specific sites. We next pondered whether differences in the sequence of P1 in γSyn (Fig. 6a) could rationalise the reduced amyloid propensity of this αSyn paralogue (Fig. 6b)^18^. γSyn is a 127 amino acid protein found in discrete populations of differentiated neurons of the peripheral and central nervous system^58^. This protein has been found in unconventional neuropathological profiles in cases of several neurodegenerative diseases^59^ and has also been reported to form amyloid aggregates in patients and animal models of amyotrophic lateral sclerosis (ALS)^60,61^ and glaucoma^62^.

The per residue aggregation propensities of αSyn and γSyn predicted in silico using Zyggregator (amyloid propensity^63^), CamSol (local solubility^64^) and ZipperDB (β-zipper propensity^65^) are shown in Supplementary Fig. 7. Both proteins have similar patterns across their sequences. For γSyn this includes an identifiable P1 (and P2) region, an aggregation-prone NAC region and an acidic, soluble, C-terminal region which is shortened by 13 residues relative to αSyn. αSyn and γSyn share 77% sequence identity in their N-terminal regions (Fig. 1a) and differ at only two positions in P1: at residue 38—which is Leu in αSyn and Met in γSyn (denoted here as L38M (αSyn_residue number_уSyn)), and at residue 42, in which Ser in αSyn is substituted with Ala in γSyn (Fig. 6a). Notably, substitution of S42 with Ala protects αSyn from amyloid formation under these experimental conditions (Fig. 5b, grey) and could also contribute to the inability of γSyn to aggregate into amyloid under the conditions explored here (Fig. 6b). Strikingly, while the substitution L38A in αSyn has little effect on its fibrillation kinetics (Fig. 5b, light blue and Fig. 6c), replacing L38 with Met (the equivalent residue from γSyn) resulted in no change in ThT fluorescence intensity on this experimental timescale (Fig. 6d), the absence of fibrils (visualised by TEM) (Fig. 6e), and the formation of oligomeric species with an average height of 3.1 ± 2.5 nm at the endpoint of aggregation (Fig. 6f, g). We therefore tested the amyloid propensity of another variant of αSyn with an aliphatic residue at position 38, L38I. Strikingly, this variant forms amyloid fibrils more rapidly than WT αSyn (compare Figs. 5b and  6h, i). Cross-seeding experiments with 10% (*w/w*) pre-formed WT αSyn fibril seeds resulted in rapid fibril growth for both L38M and L38I (Fig. 6d, h (inset)), indicating that substitution of L38 with Met disrupts amyloid nucleation, while Ile at the same site promotes this phase of assembly (as both variants efficiently elongate WT αSyn preformed fibrils (Supplementary Table 1d)). The results further substantiate the specificity of the interactions of residues in P1 in the early stages of αSyn aggregation, with Leu and Ala at residue 38 permitting aggregation, Ile accelerating aggregation, and Met at the same site resulting in few, if any, detectable amyloid fibrils under the conditions employed here (Supplementary Fig. 8).

### Sequence changes in P1 do not enable amyloid formation in γSyn

**Fig. 7:**
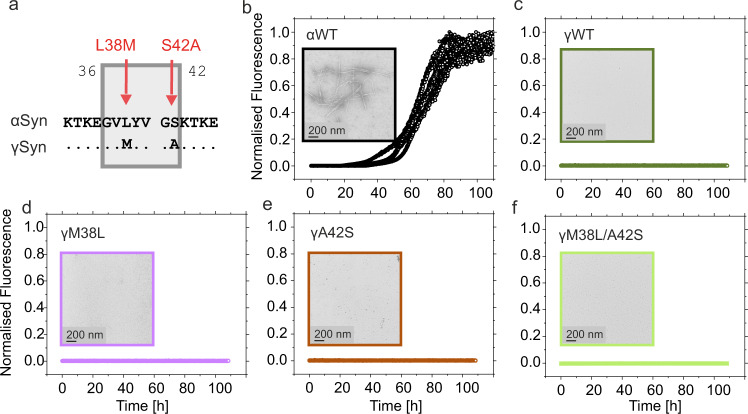
Sequence alterations in P1 do not result in aggregation of γSyn. **a** Sequence alignment of αSyn and γSyn focussing on the P1 region (boxed). Residues that differ are highlighted with red arrows and labels. Aggregation kinetics for **b** WT αSyn, **c** WT γSyn, **d** γSyn M38L, **e** γSyn A42S and **f** γSyn M38L/A42S. In each case the relevant residue in γSyn is replaced with the equivalent residue in αSyn, demonstrating that these amino acid substitutions do not induce amyloid formation under these conditions (20 mM Tris-HCl, pH 7.5, 200 mM NaCl, 37 °C, 600 rpm shaking). Seeded data (with WT αSyn seeds) are shown in Supplementary Fig. 9. The insets show representative TEM images of each sample taken at the end of the reaction (110 h) of one biological replicate (*n* = 2). % pellet are shown in Supplementary Table 1e. Source data are provided as a Source Data file.

The experiments described above show that the lag-phase of amyloid formation in αSyn can be tuned by single residue substitutions in P1 despite the presence of an unchanged NAC region, suggesting a key role of the P1 region in controlling the frequency of successful associations between monomers. As the NAC region of αSyn and γSyn are each predicted to be highly aggregation-prone, especially in the central-NAC core region (spanning residues 65–79^20,25,28^) (Supplementary Fig. 7), one possible explanation for the differences in the aggregation of these paralogues may be differences in the P1 region. Accordingly, we tested whether the γSyn variants M38L, A42S or the double substitution M38L/A42S, which replace residues in P1 of γSyn with their equivalent from αSyn (Fig. 7a) are able to promote aggregation of γSyn under the conditions employed (20 mM Tris-HCl, 200 mM NaCl, pH 7.5, 37 °C, shaking, over 110 h). While WT γSyn does not form detectable fibrils de novo over the experimental time of 110 h, and αSyn forms amyloid rapidly under these conditions, as shown here (Fig. 2a) and previously^27,66^ (Fig. 7b, c), none of the γSyn variants (M38L, A42S or M38L/A42S) assembled into detectable amyloid fibrils under these conditions in the absence (Fig. 7d–f) or presence (Supplementary Fig. 9) of preformed WT αSyn seeds. These results show that aggregation must be driven by a complex interplay of interactions of P1 residues with the NAC and/or C-terminal regions (which show only 51 and 3% sequence identity between αSyn and ySyn, respectively (Fig. 1a). Indeed, previous studies have shown that there are fewer interactions between the N- and C-termini of γSyn compared with αSyn at neutral pH^38^.

### Single residue substitutions fine-tune aggregation in vivo

**Fig. 8:**
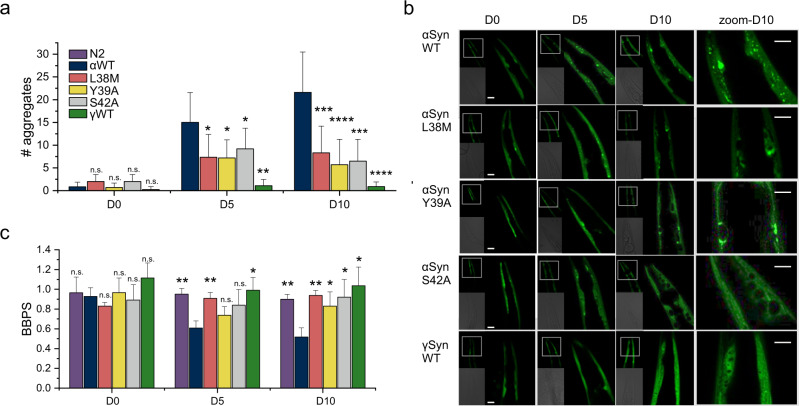
Effect of single amino-acid substitutions in αSyn and γSyn in the body wall muscle of *C. elegans*. **a** Number of inclusions (larger than ~2 µm^2^ per animal). Data shown are the mean and s.e.m. for worms (*n* = 10) that were assessed for each time point. Stars indicate significance between the number of aggregates of αSyn WT expressing worms with all other constructs. *P < 0.05; **P < 0.01; ***P < 0.001; ****P < 0.0001. A two-sided Student’s *t* test was used in all cases. Exact *p*-values are listed in Supplementary Fig. 10c. Note that for N2 worms no data were collected as they do not express YFP. **b** Confocal microscopy images (representative image of one worm from *n* = 10) showing the head region of transgenic *C. elegans* expressing WT αSyn, αSyn L38A, αSyn Y39A, αSyn S42A or WT ySyn (each fused to YFP at their C-terminus) in the body wall muscle during ageing (Day 0, Day 5 and Day 10 of adulthood). Scale bar, 10 µm. A zoomed in region is shown alongside (scale bar, 10 µm). **c** Number of body bends per second (BBPS) of N2, WT αSyn::YFP, αSyn L38M::YFP, αSyn Y39A::YFP, αSyn YS42A::YFP and WT ySyn::YFP animals from Day 0 (L4 stage), Day 5 and Day 10 of adulthood. Data shown are mean and s.e.m. for three independent experiments; in each experiment, >10 worms were assessed for each time point. **P < 0.01; *P < 0.05, a two-sided *T*-test was used. Exact p-values are listed in Supplementary Fig. 10c. Note that the N2 Bristol nematodes are used as control animals as they do not express αSyn or YFP. Western blot analysis of protein extracts isolated from N2, WT αSyn::YFP, αSyn L38M::YFP, αSyn Y39A::YFP, αSyn YS42A::YFP and WT ySyn::YFP animals using an anti-GFP antibody are shown in Supplementary Fig. 10. Source data are provided as a Source Data file for graphs in **a** and **c**.

Expressing αSyn in the body wall muscle cells of *C. elegans* (using constructs with yellow fluorescent protein (YFP) fused C-terminally to αSyn) has enabled phenotypic traits and aggregation of αSyn to be quantified in a living organism over its lifespan^40^. To determine whether the two single substitution variants of αSyn identified as protective against amyloid formation in this study (L38M and S42A) and one identified previously (Y39A)^41^ are also able to inhibit αSyn aggregation in vivo, we generated transgenic *C. elegans* strains expressing αSyn, γSyn, or the αSyn variants L38M, Y39A or S42A each fused at the C-terminus to YFP in the body wall muscle cells. Nematodes were shown to express the different variants to a similar level, as judged by western blots (Supplementary Fig. 10, see source data). The formation of puncta and nematode motility were quantified over their adult lifespans. All animals showed only a low number of inclusions at Day 0 (L4 larvae) (Fig. 8a, b), with significantly more foci observed for the nematodes expressing WT αSyn upon ageing (days 5 and 10 of adulthood) as observed previously^27,40^ (note that previous analysis of these inclusions using FRAP demonstrated that they are immobile aggregates^27^). Strikingly, nematodes expressing single substitution variants of αSyn (L38M, Y39A or S42A) showed a twofold reduction of inclusion formation at days 5 and 10 of adulthood (Fig. 8a, b). The effects of expressing these proteins in the body wall muscle cells were analysed by measuring the rate of body bends per second (BBPS) of the animals. In accord with the timing and extent of puncta formation, all nematodes were found to have similar BBPS at Day 0. While the nematodes expressing WT αSyn showed a significant decrease in body bends at Days 5 and 10, the motility of mutants expressing the αSyn variants was unaffected even at Day 10 of adulthood (Fig. 8c). For the γSyn::YFP expressing animals, visible inclusions were rarely observed, even at Day 10 (Fig. 8a) and γSyn expressing animals exhibited a similar thrashing rate (BBPS) as wild-type animals (N2 Bristol), indicating that expression of γSyn is not proteotoxic, even in aged day 10 adults (Fig. 8c), as expected from the intransigence of the protein to form amyloid in vitro (Fig. 7c).

## Discussion

More than 48 proteins are currently known to form amyloid fibrils associated with human disease^67^. Of these, 17 proteins are IDPs, or contain intrinsically disordered regions^1^. Such sequences enable dangerous liaisons since their intrinsic amyloid potential is exposed, unabridged by the protection of a native structure. This raises fundamental questions about how these initially dynamically disordered proteins self-assemble and gain order, and how this self-assembly process yields different cross-β fibril structures from similar, or identical, precursor sequences^26,68^. While many protein-protein interactions are highly specific, as exemplified by antigen-antibody recognition, virus capsid assembly and many of the protein complexes that control essential cellular reactions (such as the ribosome, nucleosomes and the nuclear pore complex)^69–71^, others are more promiscuous, as demonstrated by molecular chaperones that bind a diverse array of non-native protein clients^33,72^. Whether the initiating stages of amyloid formation rely on specific interactions, or whether these interactions are more promiscuous for αSyn (and other IDPs), however, remained unclear.

Here, we have investigated how individual residues in the P1 region (residues 36–42), that flank the essential NAC region, control αSyn self-assembly into amyloid, building on previous observations that deletion of P1 ablates aggregation of the protein at neutral pH in vitro and in *C. elegans*^27^, as does substitution of Y39 (which lies in the centre of the P1 region) with Ala^41^. The results presented reveal a remarkable specificity of the sequence of P1 in controlling the rate of αSyn aggregation, in that the sequence substitutions Y39A or S42A significantly extend the lag phase of amyloid formation at neutral pH (defined here by the lack of visible amyloid fibrils after an incubation time of 110 h), and show significantly reduced puncta and toxicity in *C. elegans*. Similar substitutions at other sites in P1 (G36A, V37A, L38A, V40A and G41A) have no effect in vitro. Perhaps even more surprisingly, while L38A has no effect on fibril growth kinetics, L38M significantly reduces fibril formation in vitro (at least on the timescale measured) and protects from amyloid-associated proteotoxicity in *C. elegans*, while L38I increased the rate of fibril formation, indicating that the sidechain-sidechain interactions involved in the initiating phase(s) of assembly are highly dependent on the identity of the amino acid at these sites. Previous investigations have also highlighted the importance of an aromatic ring at position 39 to maintain the high amyloidogenic propensity of αSyn. While Y39A reduces amyloid formation, Y39F or an αSyn variant with all Tyr residues substituted for Phe displays similar fibril formation kinetics to wild-type^55,73^. Y39F αSyn also has similar binding affinity^74^ and binding interactions^55^ to anionic liposomes and also induces similar levels of apoptosis and intracellular inclusions in dopaminergic (N27) and in human embryonic kidney 293 cells^75^ (and in H4 cells^55^). How and why these specific residue changes affect fibril formation remains unclear, requiring a more in-depth analysis of the conformational ensemble of the monomers and more information about the oligomeric species formed for each variant and the fibril structure(s) that result. Changes in the intramolecular interactions formed within the monomer caused by removing aromaticity at position 39 or altering hydrogen bonding with the sidechain hydroxyl of S42, as well as steric effects by, for example swapping L38 with a longer methionine, or L38 to the β-branched Ile, could affect amyloid propensity by changing transient secondary structure or long range/local contacts within the dynamic IDP. That these apparently subtle changes at a single site have such a dramatic effect on the lag phase of amyloid formation may also implicate specific protein-protein interactions in oligomers formed en route to fibrils. Changes in the critical concentration, or fibril stability, or alterations in the balance and efficiency of primary and secondary nucleation and/or fragmentation could also affect the observed rate of aggregation. Further experiments will be needed to define more precisely the role of each residue in atomic detail throughout the aggregation process. Further, the contribution of toxicity from oligomers versus fibrils should be investigated in more detail, especially given that the oligomers formed from S42A and Y39A either are not proteotoxic or are not formed in the *C. elegans* model used here. By contrast, the familial PD mutation. A30P has been shown to slow down fibril formation, but to also increase oligomer concentration, rationalising, at least in part, the association of this variant with early onset PD^50^.

All of the single point variants of αSyn examined here are able to elongate seeds formed from preformed WT αSyn fibrils, demonstrating that these amino acid substitutions are compatible with the WT αSyn fibril structure, as expected from the conservative nature of the substitutions, the high stability of the amyloid fold^21^, and the same sequence (residues 44–47) which can form a β-hairpin structure thought to be involved in the initiation of aggregation within the P1P2 region^76^. Interestingly, whereas αSyn fibril structures formed in vitro show cores in which no/few direct interactions with the P1 region are observed (Supplementary Fig. 11a, b)^21^, ex vivo fibrils extracted from MSA patients present a protofilament interface involving residues Y39 and V40 (Supplementary Fig. 11c)^5^. It is known that αSyn point mutations or post-translational modifications can also result in altered fibril architectures (Supplementary Fig. 11e, f)^21^. It will be interesting to explore whether the αSyn variants that were able to assemble into amyloid fibrils de novo or via seeding form new fibril morphologies. In addition, whether the variants form fibrils with different stabilities and/or ability to bind chaperones, SUMO or other biological factors, remains to be seen. Clearly, such effects could also contribute to the effects of the amino acid substitutions on amyloid formation and proteotoxicity in vivo, including in the *C. elegans* studies presented here.

Single point mutations are known to be important in the development of familial PD^50^ (Fig. 1b). Eight familial mutations have been identified to date, with two (A30P and A30G) occurring N-terminal to P1, six (E46K, H50Q G51D, A53T, A53V and A53E) found in the P2 (residues 45–57) (pre-NAC region) that juxtaposes with P1. (Note that an array of other sporadic or familial mutations associated with PD have also been reported)^77^. Interestingly, no familial mutations have yet been identified in P1, despite its now clearly demonstrated role in tuning the rate of αSyn fibril formation. This might be because such familial mutations in P1 could be rare, because changing the P1 region could be protective (rather than resulting in early onset disease), or because alterations in the P1 region result in functional defects at the synapse, despite potentially being protective against amyloid formation. Indeed, previous results have highlighted the importance of the N-terminal region, including P1 and P2 in membrane binding^27^. Consistent with the results presented here, the effect of the familial mutations on the rate of aggregation is also dependent on the specific residue involved and the nature of the sidechain introduced. For example, while the familial PD mutation A30P decreases the rate of fibril formation, the rate of aggregation of A30G is unchanged compared with WT αSyn^78^. Similarly, A53T and A53V aggregate into amyloid more rapidly than A53E^79^.

Given the known, complex, interplay of interactions between the N-terminal, NAC and C-terminal regions of αSyn in determining the properties of the ensemble of unfolded conformers that define the rate (and possibly outcome) of assembly^27,39,45^, it is not surprising that residues in other regions of αSyn can affect its rate of assembly into amyloid. For example, substitutions within the central-NAC core region (e.g. V70G/E, V74G/E or V76E/N) significantly retard aggregation^16,80^, and single point mutations within other regions of NAC (e.g. S87N^18^ and E83Q^48^) or in the C-terminal region (e.g. Y133A)^41^ have an impact on its kinetics of fibril formation. These amino acid substitutions presumably modulate the population of molecules with an exposed NAC region by subtle changes in the distribution and/or population of conformers in this dynamically disordered IDP^81,82^. Such a model is consistent with the findings presented here that the addition of the P1 sequence in trans accelerates aggregation of WT αSyn and ΔP1 by increased unfurling of the C-terminal region upon peptide binding.

Together, the results presented here suggest that the initiating stages of aggregation of αSyn involve interactions that crucially depend on the location and identity of individual sidechains at defined locations in the P1 region of this 140-residue IDP. Further experiments will be needed to define the origins of this specificity in more detail, for example by combining cross-linking, single-molecule FRET, NMR and other biophysical methods with MD simulations, to generate atomistic models of these fluctuating ensembles of monomers and early assemblies^81–83^. Other tools and approaches, such as biasing the energy landscape with small molecules added non-covalently or via tethering^84,85^, deep mutational scanning of P1 with suitable selection screens in different organisms^52,86,87^, and detailed comparison of synuclein variants such as those generated here with natural paralogues with different aggregation propensity^38,88^, may help further to tease apart these crucial interactions.

Interestingly, out of the three residues identified in the P1 region in our study to be important for controlling the rate of fibril formation (L38, Y39 and S42), Y39 has been shown to be vital to affect biological processes in other contexts such as chaperone binding^33^ and this residue is also phosphorylated in PD patients^53^. To our knowledge, L38M and S42A have not been identified as key modifiers of αSyn aggregation hitherto, and the effect of these substitutions on αSyn function at the synapse, in membrane binding and in chaperone function remain to be explored. That all of these residues are outside the highly amyloidogenic NAC region highlights the importance of analysing the interactions of such regions in more detail to develop a better understanding of the molecular mechanisms of fibril formation. This is not only important for αSyn, but for other IDPs involved in amyloid disease, wherein a common theme of disease-causing mutations occurring distal to the most aggregation-prone regions is emerging (reviewed in ref. 26). Such ‘master-controller’ regions of aggregation could form excellent targets for the development of reagents to combat amyloid formation, especially given that subtle alterations to these regions can result in dramatic changes (both acceleration and retardation) of the rate of fibril formation. The discovery of the sensitivity of the early stages of αSyn amyloid formation to the identity of individual residues in P1 region shown here offers opportunities to control amyloid assembly, by binding small molecules, chaperones, biologics, or other agents to these regions. The recent report that binding of a β-wrapin to the P1/P2 region of αSyn, and SUMO binding to P1 (or P2, but not both) prevents αSyn aggregation in vitro, in Drosophila and in neurones^31,32,35,89^ provides proof-in-principle of the potentials of such an approach.

## Methods

### Mutagenesis, expression and purification

αSyn variants containing single amino acid substitutions, deletion of, or replacement of P1 (^36^GVLYVGS^42^) by a seven-residue long Ser-Gly linker, as well as the γSyn variants M38L, A42S and M38L/A42S were generated by Q5 site-directed mutagenesis (NEB) using the WT αSyn or WT γSyn genes. Proteins were expressed recombinantly in *Escherichia coli* BL21 (DE3) and purified as described below^90^. ^15^N- and/or ^13^C-labeled protein (for NMR experiments and assignment) was expressed in HCDM1 minimal medium with ^15^N-enriched NH_4_Cl and ^13^C-enriched glucose (Cambridge Isotope Laboratories (Massachusetts, USA). Cell pellets was resuspended and homogenized in 15 mL/ litre culture lysis buffer and incubated for 30 min on a roller to disrupt the cells. Samples were then heated to 80 °C for 10 min and centrifuged for 30 min at 35,000 × *g*. 29.1 g ammonium sulfate per 100 mL lysate was added and incubated 30 min at 4 °C. The precipitated protein was pelleted by centrifuging for 30 min at 35,000 × *g* and washed with 50% (w/v) ammonium sulfate in 50 mL water and centrifuged (35,000 × *g*, 4 °C, 30 min). Finally, the protein pellet was resuspended in 300–500 mL wash buffer (20 mM Tris-HCl, pH 8.0) and loaded onto Q-sepharose column (300 mL). The protein was eluted over a 0–500 mM NaCl gradient over a volume of 500–1000 mL. αSyn containing fractions were combined and dialysed against 5 L of 50 mM ammonium bicarbonate pH 8 and lyophilised. Finally, size exclusion chromatography (HiLoadTM 26/60 Superdex 75 prep grade gel filtration column) was used with a flow rate of 2.6 mL/min. Filtered (0.22 μm) αSyn was loaded and eluted in 50 mM ammonium bicarbonate pH 8, lyophilized and stored at −20 °C. All proteins were confirmed to be >99% pure by SDS PAGE and of the correct mass using electrospray ionisation mass spectrometry (ESI-MS). Each protein was lyophilised and stored at −20 °C until use. Proteins were dissolved in the desired buffer and filtered (sterile 0.22 µm syringe filter) immediately before use.

### Synthetic peptides

Synthetic peptides were purchased from Severn Biotech Ltd. with N-terminal acetylation and C-terminal amidation. P1-peptide: Ac-KTKE-GVLYVGS-KTKE-NH_2_; P1-SG-peptide: Ac-KTKE-SGSGSGS-KTKE-NH_2_; Cys-P1-peptide (for MTSL labelling): Ac-C-KTKE-GVLYVGS-KTKE-NH_2_. For MTSL labelling, 3 mg peptide was incubated for 30 min in the presence of 5 mM DTT in 20 mM Tris HCl, 200 mM NaCl, pH 7.5. After removing access DTT using a Zeba spin column (PD10 column, GE Healthcare), the peptide sample was immediately labelled with a 40-fold molar excess of MTSL (Cambridge Isotope Laboratories (Massachusetts, USA)), reacting with the thiol group of the introduced N-terminal cysteine) for 8 h at 25 °C in 20 mM Tris-HCl, 200 mM NaCl, pH 7.5. Excess MTSL and labelled peptide were separated by reverse phase HPLC and complete modification of the peptide with MTSL was confirmed by ESI-MS (observed mass 1995.07 Da, expected mass 1995.03 Da).

### Amyloid formation monitored by ThT fluorescence

Assays were performed in 96-well flat-bottom assay plates (Corning, non-treated) sealed with a polyester SealPlate® in a FLUOstar Omega plate reader (BMG Labtech) at 37 °C with continuous shaking at 600 rpm. The experiments were performed in a volume of 100 µL and contained 100 µM αSyn or γSyn in the desired buffer and 20 µM ThT per well. Samples were measured in at least triplicate and at least two biological repeats. ThT fluorescence was excited at 444 nm and emission detected at 480 nm. In cases where the ThT signal had reached a clear plateau by the end of the experiment, the data were normalised to the maximum signal (highest value = 1). For samples which did not result in a positive ThT signal, or in which the signal had not reached a plateau, the curves were normalised to a WT αSyn which was included as a control in the same plate. Lag times and elongation rates were calculated using OriginPro software (OriginPro 2018b 64Bit) by fitting a linear gradient to the elongation phase (normalised fluorescence between 0.4 and 0.6 of the ThT-aggregation curve) where the elongation rate is the slope and the lag time is the intersection with the *x*-axis of the fitted curve. t_50_ times were calculated by GraphPad Prism 9 using a sigmoidal fit with ½ max. absorbance representing the t_50_ timepoint. Average values and standard deviation were calculated for at least three repeat measurements. To obviate the possibility of false negative results using this assay (i.e. presence of amyloid fibrils despite no increase in ThT fluorescence intensity), these data were complemented by two visualisation methods (TEM and AFM) and a pelleting assay shown to fractionate fully grown fibrils and large aggregates^43^ from soluble monomers, oligomers and small fibrils. While the latter species are not separated, the presence or absence of larger species is verified by visualisation of the contents of pelleted and soluble material using TEM and by analysis of the samples using AFM. Taken together, these assays can thus differentiate between large and small amyloid fibrils and amorphous aggregate formation.

Seeding experiments were performed as described above but using quiescent conditions. For seeding experiments, 10% (*w/w*) pre-formed WT αSyn seeds were added to the monomeric protein (100 µM). Seed preparation was performed using 500 μL of 600 μM WT αSyn in Tris-HCl pH 7.5, 20 mM NaCl stirring with a magnet stirrer at 1200 rpm at 45 °C for 48 h. The fibrils were sonicated twice for 30 s with a break of 30 s at 40% maximum power using a Cole-Parmer-Ultraprocessor-Sonicator just before adding to the sample.

### Quantification of fibril yield

The percentage of pelleted material (% pellet) was determined by SDS-PAGE, loading an unclarified sample at the experimental end point (110 h), as well as the supernatant after 30 min centrifugation at 15,500 × *g* (Microfuge SN 100/90). Gels were stained with InstantBlue® Coomassie Protein Stain and imaged on an Alliance Q9 Imager (Uvitec). Band intensities were quantified using ImageJ 1.52a.

### Negative stain TEM

End-point samples from ThT assays (usually after 110 h for de novo growth and 40 h for seeding experiments) were diluted fivefold with 18 MΩ H_2_O. A sample (5 µL) was loaded onto a carbon-coated copper grid (provided by the EM facility, University of Leeds) and incubated for 20 s before drying with filter paper. The grid was washed three times with water in a drop wise fashion with drying steps in-between each wash. Fibril samples were then stained twice with 1% (w/v) uranyl acetate, blotted as before and imaged on a FEI Tecnai T12 electron microscope.

### Far UV CD

Far UV CD spectra of peptides P1 and P1-SG (20 µM, 20 mM Tris-HCl, pH 7.5, 200 mM NaCl) were acquired in quartz cuvettes (Hellma) with 1 mm path length, using a 2 nm bandwidth, 1 s time step and 1 nm increments at 25 °C using a ChirascanTM plus CD Spectrometer (Applied Photophysics). Three scans ranging from 190 to 260 nm were measured for each sample and averaged (Microsoft Excel 2013).

### NMR backbone assignments of WT αSyn and ΔP1 at pH 7.5

WT and ΔP1 αSyn variants were ^13^C and ^15^N labelled for NMR backbone assignment purposes. 200 μM protein in 20 mM Tris-HCl, 20 mM NaCl, 10% (v/v) D_2_O, 0.02% (w/v) sodium azide, pH 7.5 was used and experiments were performed at 15 °C to acquire triple-correlation experiments: HNCO, HNcaCO, HNCACB, HNcoCACB, HNN-TOCSY, hNcaNNH and hNcacoNNH. All experiments were acquired using non-uniform sampling, where just 35% of sparse data were recorded on a Bruker AVANCE III 950 MHz spectrometer equipped with a triple-resonance TCI (3 mm) cryoprobe. NMR data processing and spectra reconstruction were performed using NMRpipe, and data analysis was performed using the ccpNMR-Analysis software. HN, Cα, Cβ and CO chemical shifts were deposited at Biological Magnetic Resonance Bank (BMRB) with accession numbers 51120 and 51121 for WT αSyn and ΔP1, respectively. For αSyn WT at pH 4.5 previous assignments (BMRB 27900) were used^27^.

### NMR spectroscopy

For all NMR experiments in the presence of peptide P1 or P1-SG, ^1^H-^15^N HSQC spectra were obtained using 100 µM ^15^N spin-labelled αSyn in 20 mM Tris-HCl, pH 7.5, 200 mM NaCl, 15 °C (note that aggregation does not occur at this temperature in the quiescent NMR tube, as shown by measurement of chemical shifts and intensities after data acquisitions of up to 65 h). For chemical shift perturbation analysis 0, 500 µM or 1 mM peptide (P1-peptide or P1-SG peptide) was added and data acquired using a Bruker AVANCE III 750 MHz spectrometer. Spectra were processed in Topspin (Bruker). Peak positions and intensities were extracted using ccpNMR-analysis, and HN-CSP were calculated using Eq. (1):

1 $$\Delta \delta=\sqrt{{(5 \,*\, {\delta }^{1}{\rm H})}^{2}+{({\delta }^{15}{\rm N})}^{2}}$$

For the comparison of chemical shifts at different pH values, 100 µM αSyn in 20 mM Tris-HCl, pH 7.5, 20 mM NaCl or in 20 mM sodium acetate, pH 4.5, 20 mM NaCl were measured, and peak positions analysed as described above.

For PRE NMR experiments, 100 µM ^15^N spin-labelled protein with 100 µM ^14^N MTSL labelled peptide was used. The diamagnetic spectra were acquired 30 min after adding 1 mM ascorbic acid. Data were collected using a Bruker AVANCE III 950 MHz spectrometer and data were processed as described above, the peak heights being used to calculate intensity ratios (paramagnetic/ diamagnetic).

### Native nESI-mass spectrometry

WT αSyn and ΔP1 αSyn samples with a final concentration of 20 μM were prepared in 20 mM aqueous ammonium acetate buffer (pH 7.5). The P1 peptide was diluted into the buffer solution to achieve a final molar ratio of αSyn and P1-peptide of 1:10. Native ESI-MS analysis was performed on a Synapt G1 HDMS instrument (Waters Corp., Wilmslow, UK). All samples were analysed using positive ionisation ESI with a spray capillary voltage of 1.2 kV. The following instrumental parameters were used: source temperature 30 °C; sampling cone 30 V; backing pressure 2.25 mbar; extraction cone 1 V; trap collision energy 5 V; trap DC bias 30 V; transfer collision energy 2 V. The system was calibrated with NaI cluster ions from a 2 μg/μL 50:50 2-propanol:water solution. Data were acquired over the m/z range of 100-4000 and processed by using MassLynx V4.1 supplied with the mass spectrometer. CID MS/MS experiments were conducted in the trap cell of the Synapt G1 mass spectrometer with argon gas, the collision energy was applied increasingly to the trap cell from 5 to 60 V.

### AFM

Mica was freshly cleaved before being treated to create a positive surface charge by adding poly-l-lysine (70–150 kDa) at 15 µg/mL for 10 s followed by drying with nitrogen. A sample volume of 90 μL of protein (WT αSyn, L38M, Y39A or S42A) was taken at the end point of a fibril growth assay (as described above) before being deposited at a concentration of 30 μM onto poly-l-lysine treated mica and allowed to incubate for 4 min. The mica surface was then rinsed with buffer (50 mM sodium phosphate buffer, 300 mM KCl, pH 7.5) via fluid exchange, maintaining the samples in a liquid environment. AFM observations were performed in liquid in tapping mode using a Dimension FastScan Bio with FastScan-D-SS probes (Bruker) in the same buffer. The force applied by the tip on the sample was minimized by maximizing the set point whilst maintaining tracking of the surface. Heights of single particles were measured automatically using routines written in MATLAB (https://github.com/George-R-Heath/Particle-Detect). Heights and lengths of fibrils were measured either automatically using MATLAB (https://github.com/George-R-Heath/Correlate-Filaments) or manually in ImageJ for densely packed overlapping fibrils.

### Maintenance and generation of transgenic *C. elegans* strains

The WT αSyn gene was fused at its C-terminus to YFP in vector pPD30.38 kindly provided by the Nollen lab^40^. The WT γSyn gene (purchased from Eurofins) was ligated into the worm-vector via AgeI and NheI restriction sites. The αSyn and уSyn genes were mutated to contain the relevant single point substitutions using PCR (Q5 mutagenesis kit). The resulting constructs were microinjected at a concentration of 30 ng/μL into the gonad of N2 Bristol (CGC (Caenorhabditis Genetics Centre, University of Minnesota)). Transgenic *C. elegans* expressing each construct were then generated by microinjection into the germline of N2 nematodes, resulting in strains PVH250 *pccEx023[unc-54p::a-synucleinL38M::YFP]*, PVH251 *pccEx024[unc-54p::a-synucleinY39A::YFP]* and PVH252 *pccEx025[unc-54p::a-synucleinS42A::YFP]* (Nemametrix). Nematodes expressing WTαsyn::YFP were created using gene bombardment and kindly provided by Ellen Nollen^40^.

### Western blot analysis of protein expression and aggregation assays in nematodes

Nematodes were collected from plates, washed in M9 buffer, and resuspended in lysis buffer (20 mM Tris-HCl, pH 7.5; 10 mM β-mercaptoethanol; 0.5% (v/v) Triton X-100; supplemented with complete protease inhibitor (Roche)) before shock freezing in liquid nitrogen. Three freeze−thaw cycles were performed before the worm pellet was ground with a motorised pestle and lysed on ice. The lysate was centrifuged at 1000 *×* *g* for 1 min in a table-top centrifuge to pellet the carcasses. Protein concentration was determined using a Bradford assay (ThermoFisher). Samples were then mixed 1:1 with SDS loading buffer (2% (w/v) SDS, 10 % (v/v) glycerol, 0.1 % (w/v) bromophenol blue, 100 mM DTT), then boiled for 10 min, and 7.5 μg of protein was loaded onto a 4–20% gradient Tris HCl gel (Bio-Rad). Protein bands were blotted onto a PVDF membrane, and synuclein::YFP and tubulin (control) were visualised using a mouse anti-GFP antibody (anti-GFP (1:1000) (BioLegend clone B34, 902601)) or mouse anti-tubulin antibody (1:5000) (Sigma clone DM1A monoclonal, T9026), followed by an anti-mouse horseradish peroxidase-coupled secondary antibody (1:5000) (Cell Signalling Technology, 7076S). Bands were visualized using the Clarity^TM^ ECL Western Substrate (Bio-Rad). Images of uncropped and unprocessed scans are available as source data file.

Imaging and motility experiments with *C. elegans* were performed as follows. For imaging, *C. elegans* was cultured on NGM plates seeded with *E. coli* HT115 cells at 20 ^o^C^27^. *C. elegans* were imaged using a Zeiss LSM880 confocal fluorescent microscope through a 40 × 1.0 numerical aperture objective with a 514 nm line for excitation of YFP. Before imaging, age-synchronised animals at different development stages (Day 0 (L4 stage), Day 5 and Day 10) were treated with 5 mM sodium azide solution and mounted on 2% (w/v) agar pads. The number of αSyn::YFP foci were then counted from the tip of the head region to the end of the pharyngeal terminal bulb. All fluorescence spots larger than ~2 µm^2^ were considered puncta.

To determine motility of the worms, a total of 10 age-synchronised animals were used for each assay and each experiment was repeated at least three times. Animals were moved into M9 buffer at indicated time points (Day 0, 5, and 10 of adulthood) and thrashing rates were measured by counting body bends for 15–30 s using the wrMTrck plugin for ImageJ (available at http://www.phage.dk/plugins/wrmtrck.html)^91^. Error bars represent SEM of three biological replicates.

### In silico methods to determine aggregation propensity

The aggregation propensity, solubility and ability to form a steric zipper were analysed by using the online tools Zyggregator^63^, Camsol^64^ and ZipperDB^65^ at pH 7.0.

### Reporting summary

Further information on research design is available in the Nature Research Reporting Summary linked to this article.

## Supplementary information

Supplementary Information

Reporting Summary

## Data Availability

The NMR Chemical shift assignments can be accessed using BMRB accession numbers. BMRB 51120(WT-αSyn, pH 7.5) and BMRB 51121 (ΔP1 αSyn, pH 7.5). All other data generated in this study are provided in the Supplementary Source Data file. (University of Leeds Data Repository: (10.5518/1051)). Source data are provided with this paper.

## Source data

Source Data

## Code availability

MATLAB codes used to analyse AFM images are freely available in the following GitHub repositories for single particle height analysis (George-R-Heath/Particle-Detect: Particle-Detect (v1.0.0). Zenodo. 10.5281/zenodo.6946146) and fibril length and height analysis (George-R-Heath/Correlate Filaments: Correlate Filaments (v1.0) Zenodo. 10.5281/zenodo.6945954.
