# Supplementary material for: Single residue modulators of amyloid formation in the N-terminal P1-region of α-synuclein
Source: Nat Commun. 2022 Aug 25;13:4986. doi: 10.1038/s41467-022-32687-1 (PMC9411612; doi:10.1038/s41467-022-32687-1)
Supplement: Supplementary file 1 — Supplementary Information [file 41467_2022_32687_MOESM1_ESM.pdf]

SUPPLEMENTARY INFORMATION

Single residue modulators of amyloid formation in the N-terminal P1-region of α-synuclein

Sabine M. Ulamec[1], Roberto Maya-Martinez[1], Emily J. Byrd[1], Katherine M. Dewison[1], Yong Xu[1], Leon F. Willis[1], Frank Sobott[1], George R. Heath[2], Patricija van Oosten Hawle[1], Vladimir L. Buchman[3], Sheena E. Radford[1,*], David J. Brockwell[1,*]

Supplementary Tables

Supplementary Table 1

a						
Figure 2	αWT, pH 4.5	αWT, pH 7.5	ΔP1, pH 4.5	ΔP1, pH 7.5	P1-SG-αSyn, pH 4.5	P1-SG-αSyn, pH 7.5
Lag time [h]	2.0 ± 0.3	48.9 ± 0.9	18.4 ± 0.8	-	21.2 ± 1.4	-
Elongation rate*	0.7 ± 0.5	0.2 ± 0.05	2.0 ± 0.2	-	1.3 ± 0.2	-
t_{50} [h]	5.0 ± 1.0	67.6 ± 3.3	20.8 ± 1.4	-	29.1 ± 10.1	-
% pellet	100	65	95	5	60	5

b					
Figure 3	WT: peptide P1 1:0	WT: peptide P1 1:1	WT: peptide P1 1:10	WT: peptide P1-SG 1:1	WT: peptide P1-SG 1:10
Lag time [h]	48.6 ± 3.6	30.6 ± 2.2	27.4 ± 1.8	74.8 ± 7.7	91.7 ± 8.1
Elongation rate*	0.38 ± 0.07	0.7 ± 0.1	3.1 ± 2.1	0.6 ± 0.1	1.0 ± 0.3
t_{50} [h]	51.3 ± 2.2	40.1 ± 7.2	26.6 ± 3.9	81.6 ±9.2	80.0 ± 10.9
% pellet	65	40	65	65	15
	ΔP1: peptide P1 1:0	ΔP1: peptide P1 1:1	ΔP1: peptide P1 1:10	ΔP1: peptide P1-SG 1:1	ΔP1: peptide P1-SG 1:10
Lag time [h]	-	-	11.9 ± 0.1	-	30.6 ± 1.1
Elongation rate*	-	-	2.2 ± 0.4	-	0.9 ± 0.04
t_{50} [h]	-	-	14.4 ± 0.7	-	37.0 ±2.5
% pellet	0	0	70	0	50
	ΔΔ: peptide P1 1:0	ΔΔ: peptide P1 1:1	ΔΔ: peptide P1 1:10	ΔΔ: peptide P1-SG 1:1	ΔΔ: peptide P1-SG 1:10
Lag time [h]	-	-	16.3 ± 1.8	-	-
Elongation rate*	-	-	2.9 ± 0.5	-	-
t_{50} [h]	-	-	20.1 ± 1.5	-	-
% pellet	5	10	90	5	5

C								
Figure 5	WT	G36A	V37A	L38A	Y39A	V40A	G41A	S42A
Lag time [h]	65.0 ± 4.0	72.6 ± 4.8	40.2 ± 3.4	56.7 ± 6.7	-	42.5 ± 4.2	53.6 ± 3.5	-
Elongation rate*	0.38 ± 0.07	0.51 ± 0.1	0.33 ± 0.09	0.30 ± 0.07	-	0.24 ± 0.03	0.41 ± 0.03	-
t_{50} [h]	79.8 ± 6.0	83.3 ± 4.0	57.5 ± 7.2	74.6 ± 10.0	-	61.8 ± 5.5	66.1 ± 3.0	-
% pellet	65	65	75	75	25	40	65	10

d						
Figure 6	γWT	L38A	L38M	L38M, seed	L38I	L38I, seed
Lag time [h]	-	56.7 ± 6.7	-	0	28.1 ± 3.6	0
Elongation rate*	-	0.30 ± 0.07	-	2.3 ± 0.4	0.2 ± 0.04	1.5 ± 0.1
t_{50} [h]	-	74.6 ± 10.0	-	1.4 ± 0.1	44.3 ± 5.8	1.9 ± 0.08
% pellet	0	75	40	90	35	75

e			
Figure 7	γM38L	γA42S	γM38L/A42S
Lag time [h]	-	-	-
Elongation rate*	-	-	-
t_{50} [h]	-	-	-
% pellet	0	0	0

Table S1: Lag times, elongation rates, t_{50} and % pellet for αSyn variants. Measured parameters correlate to Figure 2, Figure 3, Figure 5, Figure 6 and Figure 7 of the main text. The rates of aggregation in each condition were measured in at least triplicate measurements and two biological repeats. The errors show the standard deviation of the mean of the replicates. No aggregation after 100 h incubation is indicated by "-". Given errors in estimating % pellet via SDS PAGE subsequent to centrifugation (see Methods) the values were rounded to the nearest 5%. *units of elongation rate are 10*[RFU/h]. All experiments were carried out using 100 μM αSyn, pH 7.5, 200 mM NaCl, 37°C, 600 rpm unless stated otherwise.

Supplementary Table 2

a								
**Supp Fig 6**	**WT**	**G36A**	**V37A**	**L38A**	**Y39A**	**V40A**	**G41A**	**S42A**
Lag time [h]	0	0	0	0	0	0	0	0
Elongation rate*	2.1 ± 0.2	2.9 ± 1.0	0.1 ± 0.07	0.87 ± 0.08	2.2 ± 0.2	0.52 ± 0.04	1.7 ± 0.1	0.6 ± 0.008
t_{50} [h]	1.5 ± 0.1	1.5 ± 0.5	4.8 ± 0.3	3.6 ± 0.3	1.5 ± 0.2	9.2 ± 2.3	2.4 ± 0.1	4.4 ± 0.6
% pellet	85	75	80	90	85	50	85	85

b		
**Supp Fig 5**	**ΔP1, seed**	**P1-SG-αSyn, seed**
Lag time [h]	-	-
Elongation rate*	-	-
t_{50} [h]	-	-
% pellet	20	20

c				
**Supp Fig 9**	**yWT, seed**	**yM38L, seed**	**yA42S, seed**	**yM38L/A42S, seed**
Lag time [h]	-	-	-	-
Elongation rate*	-	-	-	-
$t_{1/2}$	-	-	-	-
% pellet	10	5	5	15

Table S2: Lag times, elongation rates, t_{50} and % pellet of αSyn variants. Measured parameters correlate to Supplementary Figure 6 and Supplementary Figure 9. The rates of aggregation in each condition were measured in at least triplicate measurements and two biological repeats. The errors show the standard deviation of the mean of the replicates. No aggregation after 100 h incubation (or 42 h for seeded experiments) is indicated by "-". Given errors in estimating % pellet via SDS PAGE subsequent to centrifugation (see Methods), the values were rounded to the nearest 5%. *The elongation rate is in units of 10*[RFU/h]. All experiments were carried out using 100 μM αSyn, pH 7.5, 200 mM NaCl, 37°C, 600 rpm unless stated otherwise.

Supplementary Table 3

Variant	Fibril Length [nm]	Fibril Height [nm]	Oligomer Height [nm]	Monomer height [nm]	Number measured (n)
**WT**	106 $\pm$ 73 (n = 232)	13.6 $\pm$ 3.4 (100%)	- (0%)	- (0%)	232
**Y39A**	170 $\pm$ 115 (n = 27)	13.5 $\pm$ 3.8 (1.1%)	4.3 $\pm$ 3.3 (61%)	1.6 $\pm$ 1.0 (38%)	2,355
**S42A**	153 $\pm$ 74 (n= 135)	14.6 $\pm$ 2.0 (15%)	3.9 $\pm$ 2.1 (65%)	2.5 $\pm$ 0.8 (20%)	898
**L38M**	-	- (0%)	2.6 $\pm$ 1.7 (45%)	1.4 $\pm$ 0.8 (55%)	2,502

Table S3: Physical properties of aggregates imaged by AFM. Fibril lengths are averages with errors showing the standard deviation of the mean (n = number of fibrils measured). Height and height errors show the peak position and standard deviation of Gaussian fitting to height histograms. % values give proportion of each feature. – indicates no particles of that type were observed.

Supplementary Figures

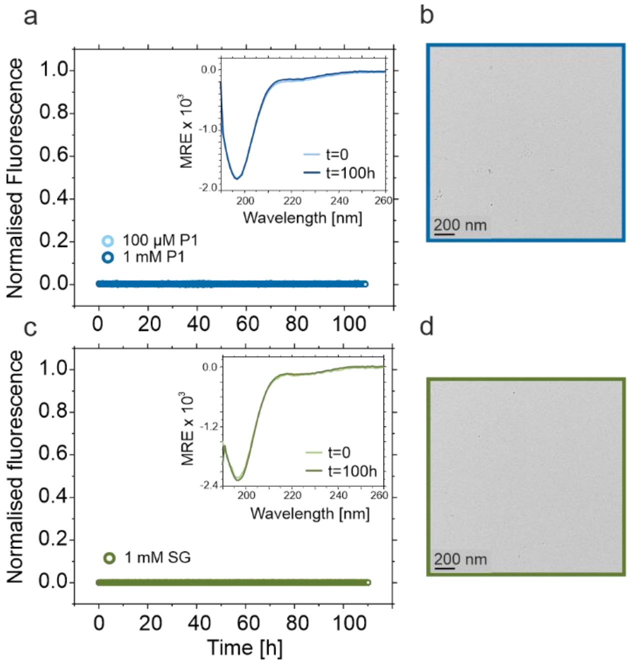

Supplementary Figure 1: The synthetic peptides P1 and P1-SG do not aggregate in isolation under the conditions employed. Analysis of (a,b) the P1 peptide or (c,d) the peptide P1-SG in isolation (100 µM or 1 mM (pH 7.5, 200 mM NaCl, 37°C, 600 rpm)) by ThT fluorescence (n=2 biological replicates) (a,c), far UV CD (inset) and negative stain TEM (b,d) shows that the peptides do not aggregate in isolation under the conditions employed (n=1). Note: data points for 100 µM and 1mM P1 peptide overlay in (a). Source data are provided as a Source Data file.

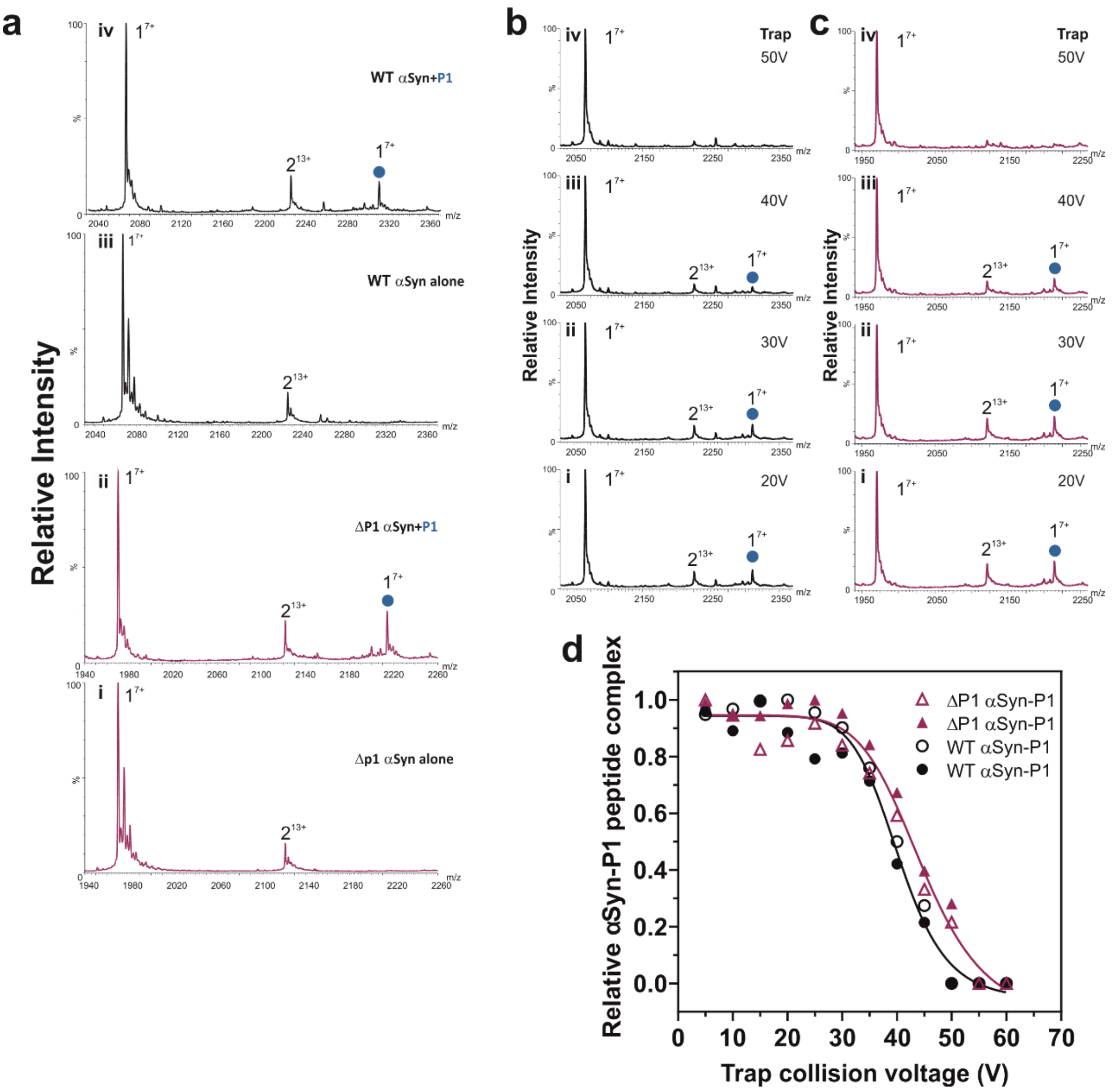

Supplementary Figure 2: The interaction between WT αSyn, ΔP1 and the P1 peptide characterised by native nESI-MS. (a) Positive-ion nESI mass spectra of (i) ΔP1 αSyn alone, theoretical mass 13784 Da; (ii) ΔP1 αSyn with added P1 peptide (theoretical mass 1708 Da) at a molar ratio of ΔP1 αSyn:P1 peptide of 1:10; (iii) WT αSyn alone, theoretical mass 14460 Da; (iv) WT αSyn with added P1 peptide at a molar ratio of WT αsyn:P1 peptide of 1:10. 1:1 peptide:protein binding is denoted with a blue circle. (b) ESI mass spectra obtained of the WT αSyn-peptide P1 complex at different trap cell voltages; (i) 20 V; (ii) 30 V; (iii) 40 V; (iv) 50 V performed through collision induced dissociation (CID). (c) as (b), but for the ΔP1 αSyn-peptide P1 complex. (d) Collison induced dissociation of WT αSyn-peptide P1 complex and ΔP1 αSyn-peptide P1 complex with the intensity of the complex relative to the sum of the bound and unbound intensities plotted versus the trap cell voltage. The experiment was performed in duplicate (as indicated in the inset legend) and the data fitted to a sigmoid function (solid line). Source data are provided as a Source Data file.

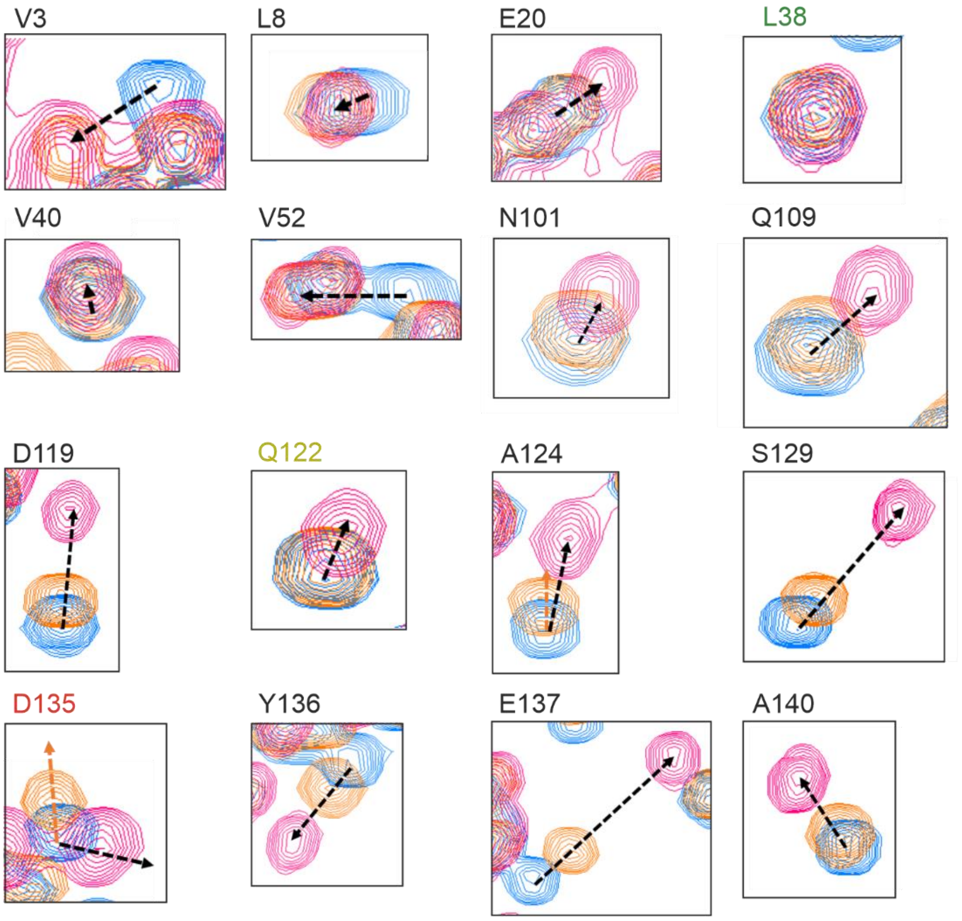

Supplementary Figure 3: Cross-peaks of ^{1}H-^{15}N HSQC spectra to identify differences between pH titration and P1-peptide addition. Peaks for WT αSyn in 20 mM Tris HCl pH 7.5 (blue), αSyn WT in 20 mM sodium acetate pH 4.5 (pink) (both with 200 mM NaCl)and αSyn WT with 10x P1 peptide (orange). Black arrows indicate direction of HN-CSPs. Each box is labelled with the corresponding residue, black residue label: changes occur along a similar vector, green residue label: no change for any of the conditions, yellow: no change for one of the conditions but a clear CSP for the other, red: distinct HN-CSPs for the pH titration and peptide addition. These data indicate that similar, but not identical, conformational changes occur under the two conditions.

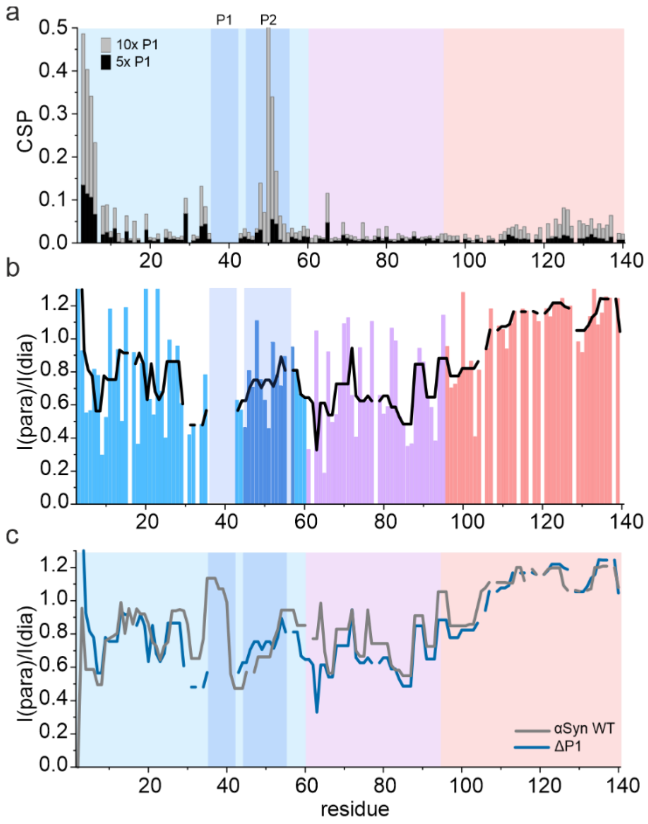

Supplementary Figure 4: Binding of peptide P1 alters the conformational ensemble of ΔP1. (a) HN-CSPs and (**b**) ^{1}H-^{15}N PRE effects upon addition of peptide-P1 to 100 μM ^{15}N-labelled ΔP1 (pH 7.5, 200 mM, 15°C). The black line represents the median value over a rolling window of 5 residues (here using equimolar peptide P1:ΔP1. (**c**) Comparison of the median value PREs of WT αSyn and ΔP1 upon the addition of equimolar P1-peptide are compared to highlight their similar profiles. Note that residues 36-42 are deleted in ΔP1. The N-terminal region is shaded light blue, NAC is in purple and the C-terminal region in red. The P1 and P2 regions are highlighted in darker blue. Source data are provided as a Source Data file.

100 nm

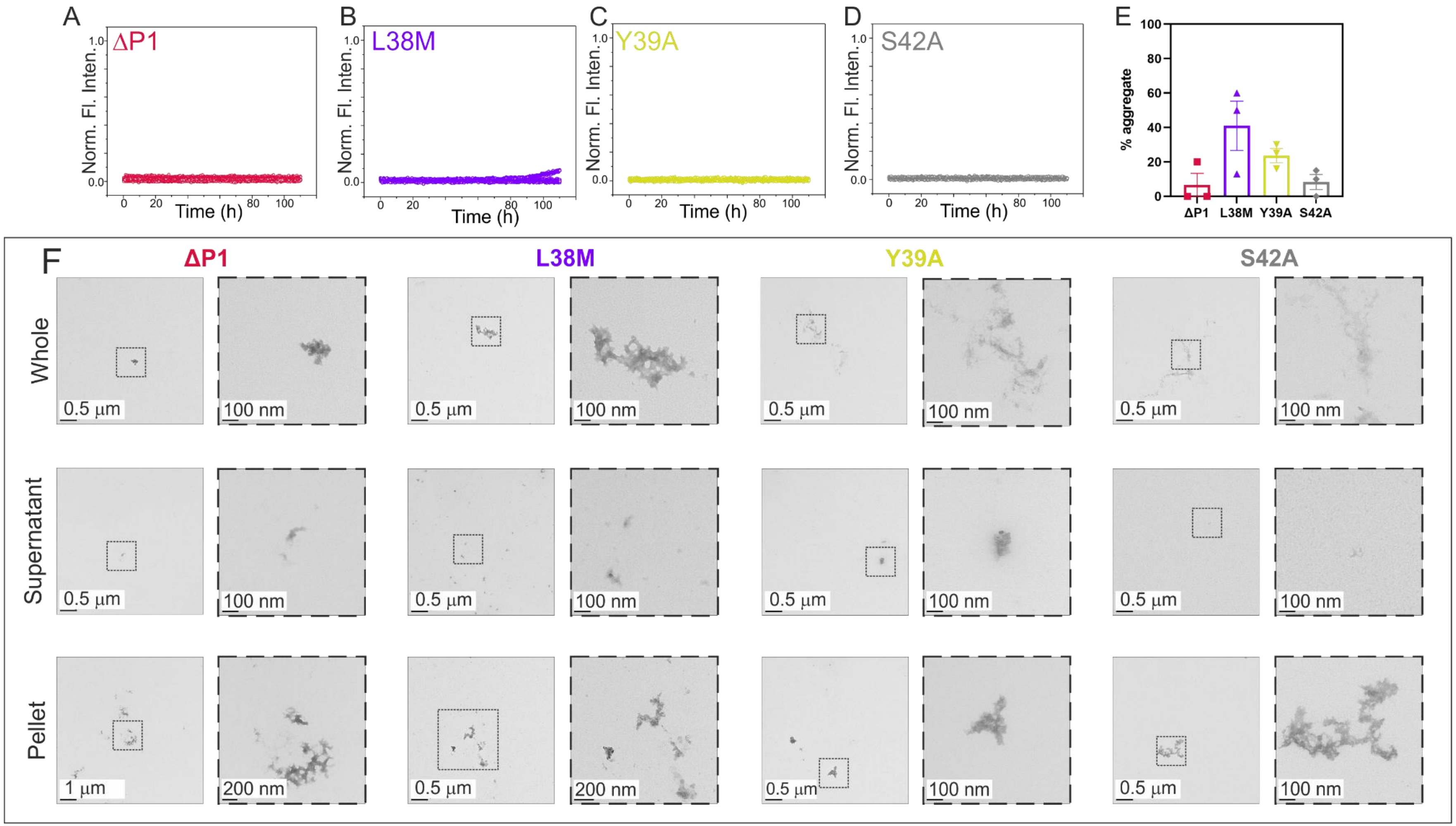

Supplementary Figure 5: Quantification and characterisation of material fractionated in the pelleting assay for variants with reduced aggregation propensity. (A-D) ThT fluorescence intensity kinetic profiles for ΔP1, L38M, Y39A and S42A αSyn over 110 h. (E) % protein of an end-point sample that is found in the pellet after bench top centrifugation at 13000 rpm for 30 mins. Bars are the average of three biological repeats, errors are the standard error of the mean. (F) TEM imaging of samples before/after centrifugation. For each variant, wide (9,300x or 11,000x magnification) and magnified (30,000x or 49,000x magnification) views are of a representative image chosen from one biological repeat shown before centrifugation (“whole”) and after (clarified solution is “supernatant”, pellet resuspended in the same volume as the supernatant is “pellet”). Source data are provided as a Source Data file.

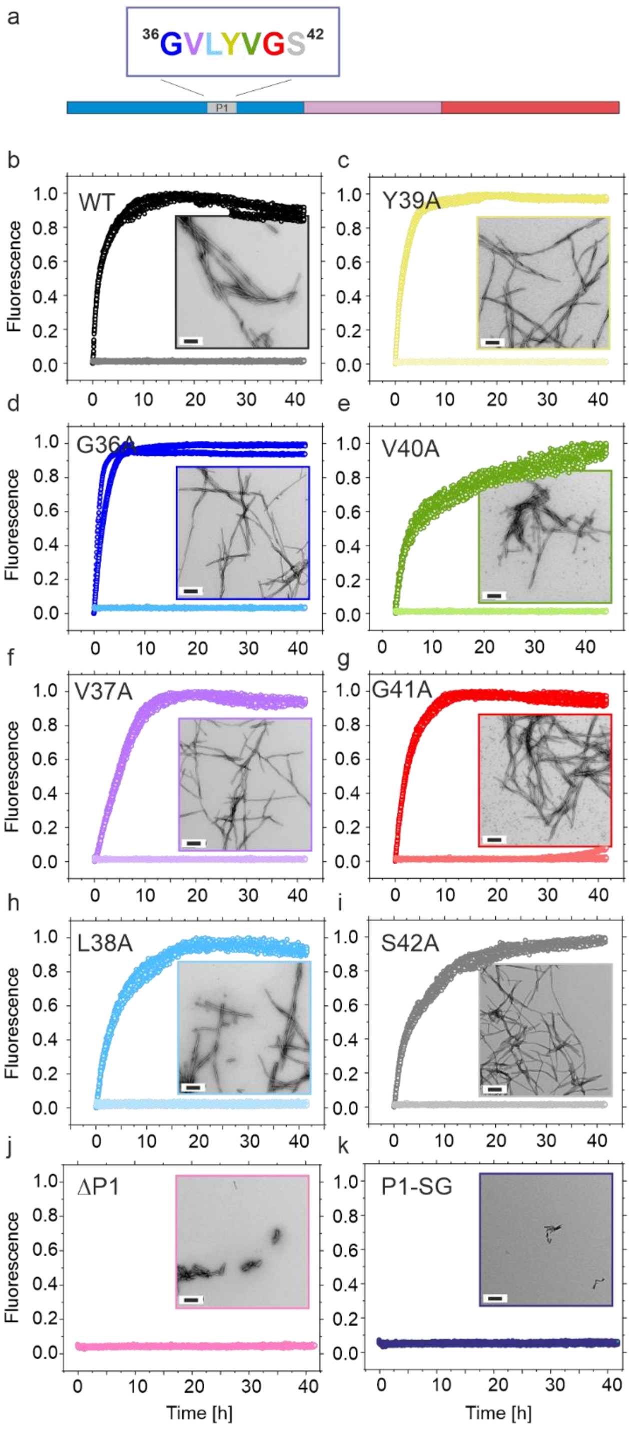

Supplementary Figure 6: Cross-seeding αSyn variants with pre-formed fibrils of WT αSyn. (a) Schematic of αSyn showing the sequence of P1. (b-k) The ability of the different αSyn variants to elongate seeds formed from WT αSyn at pH 7.5 was assessed using ThT fluorescence. For each sample 10% (*w/w*) preformed fibril seeds of WT αSyn formed at pH 7.5 (Methods) were added to monomers (100 μM) of each variant and fibril growth was monitored at pH 7.5 (200 mM NaCl, 37°C), quiescent. Fibril growth was monitored in the presence (dark colours) or absence (light colours) of fibril seeds. Representative TEM images (selected from replicate experiments) after each seeding experiment (42 h) are shown inset (scale bar 200 nm). Note that the data points with/without seeding in (j) and (k) overlay. Lag times, elongation rate, t_{50} values and % pellet are shown in Supplementary Table 2a,b. Source data are provided as a Source Data file

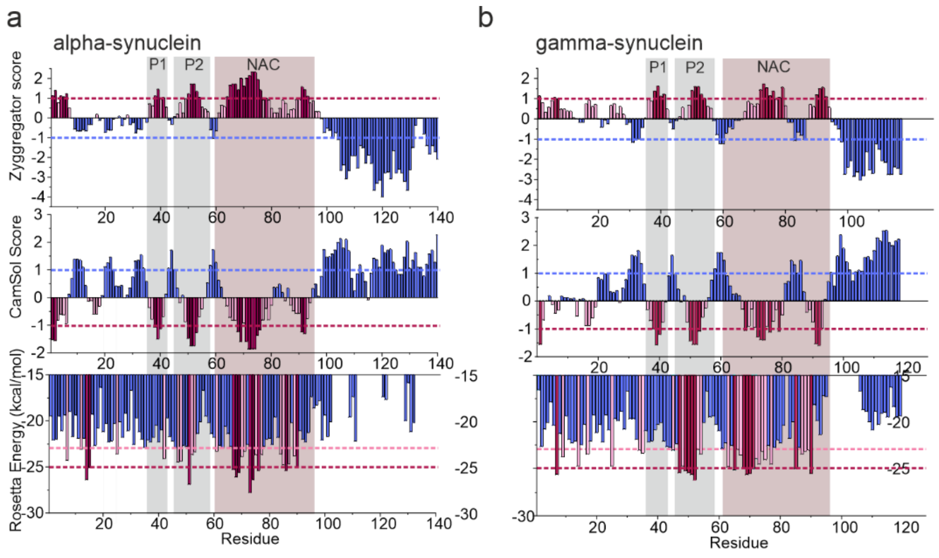

Supplementary Figure 7: *In silico* analysis of the α- and γ-synuclein sequences. Zyggregator[62], Camsol[63] and ZipperDB[64] profiles for (**a**) αSyn and (**b**) γSyn. Red bars indicate aggregation-prone/low solubility regions. Pink bars indicate residues with a higher than average aggregation propensity/low solubility, but which do not meet the threshold and blue bars represent residues with low aggregation propensity. Red dashed lines indicate the low solubility/high aggregation propensity threshold, while blue dashed lines show threshold values for high solubility/low aggregation propensity. For Zipper DB, the red/pink dashed lines show the threshold value of residues with a high probability of β-zipper formation[64]. P1 and P2 region are highlighted in grey and NAC region in pale red. Data for (**a**) are redrawn from[27]. Source data are provided as a Source Data file.

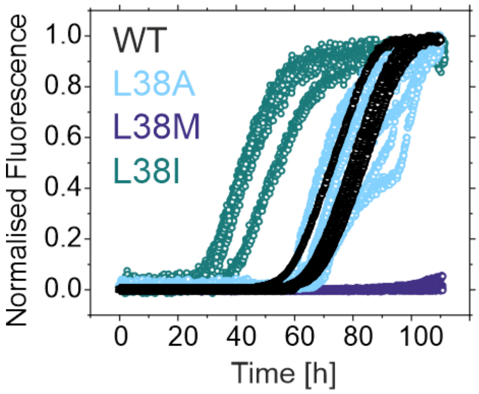

Supplementary Figure 8: Overlay of ThT aggregation assays of αSyn L38X variants. Direct comparison of the aggregation kinetics of WT αSyn (black), L38A (light blue), L38M (dark blue) and L38I (green) variants of αSyn using 100 µM protein, 20 mM Tris-HCl, pH 7.5, 200 mM NaCl, 37°C, 600 rpm. Data are reproduced from Figures 5 and 6 to enable their direct comparison. Source data are provided as a Source Data file.

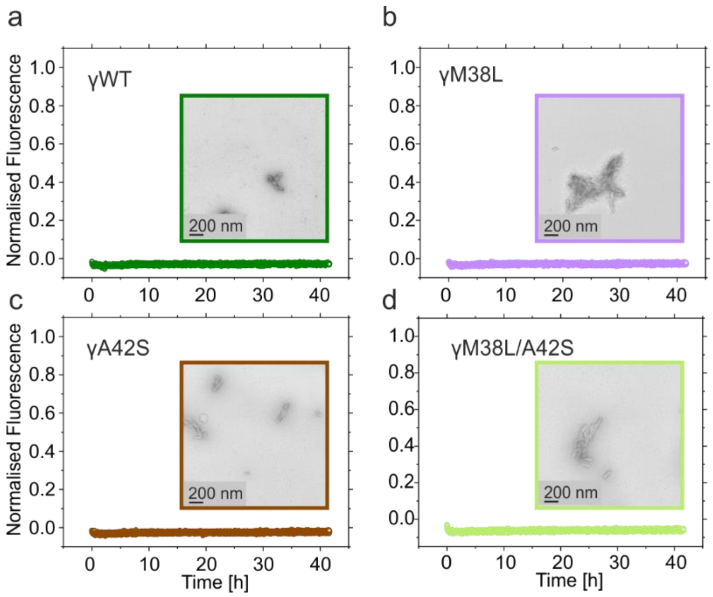

Supplementary Figure 9: Cross-seeding γSyn constructs with 10% (*w/w*) preformed αSyn WT seeds does not result in fibril formation. Aggregation kinetics of (a) WT γSyn, (b) γSyn M38L, (c) γSyn A42S and (d) γSyn M38L/A42S seeded with 10% (*w/w*) seeds of preformed WT αSyn fibrils formed at pH 7.5, 200 mM NaCl. Each experiment was performed using 100 µM γSyn at pH 7.5 (200 mM NaCl, 37°C) quiescent. The inserts show representative (chosen from replicate experiments) negative stain TEM images taken at the endpoint of one experiment (42 h). % pellet are shown in Supplementary Table 2c. Source data are provided as a Source Data file.

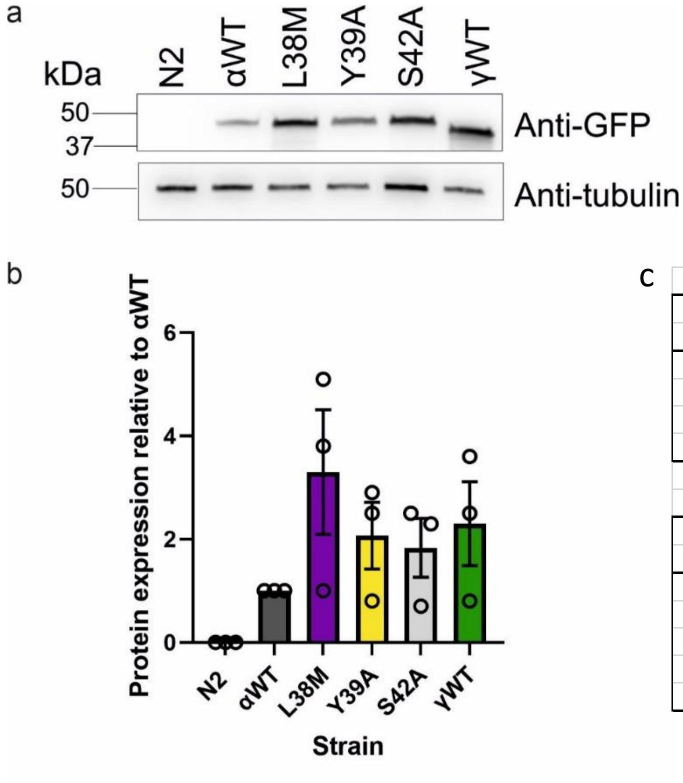

c

	Aggregates		
αSyn WT vs	D0	D5	D10
**L38M**	0.0588 ns	0.0118 *	0.0007 ***
**Y39A**	0.712 ns	0.0101 *	<0,0001 ****
**S42A**	0.0948 ns	0.0446 *	0.0001 ***
**γSyn**	0.1215 ns	0.0015 **	<0,0001 ****

	Body bends		
αSyn WT vs	D0	D5	D10
**L38M**	0.1797 ns	0.0058 **	0.0056 **
**Y39A**	0.7228 ns	0.1201 ns	0.042 *
**S42A**	0.7424 ns	0.109 ns	0.0409 *
**γSyn**	0.1595 ns	0.0188 *	0.0254 *
**N2**	0.7538 ns	0.0037 **	0.0077 **

Supplementary Figure 10: Western Blot analysis of expression level of synuclein::YFP levels in different *C. elegans* strains. (a) Western blot using anti-GFP to visualise synuclein expression levels. Tubulin was used as a loading control. The loading control (anti-tubulin) was run on a different gel and membrane loaded with the same protein sample and treated and analysed in an identical manner (necessitated by the similar mass of synuclein:YFP and tubulin). The images are cropped, showing all relevant bands. (b) Densitometry of three biological replicates of the western blot analysis. Mean expression levels are normalised (by densitometry) to those of WT αSyn (αWT). Error bars show the standard deviation over the three biological replicates. Source data are provided as a Source Data file. (c) exact P-values for two-sided Student's t test for worms expressing WT αSyn against listed construct for number of aggregates (Figure 8a) and body bends per second (Figure 8c)

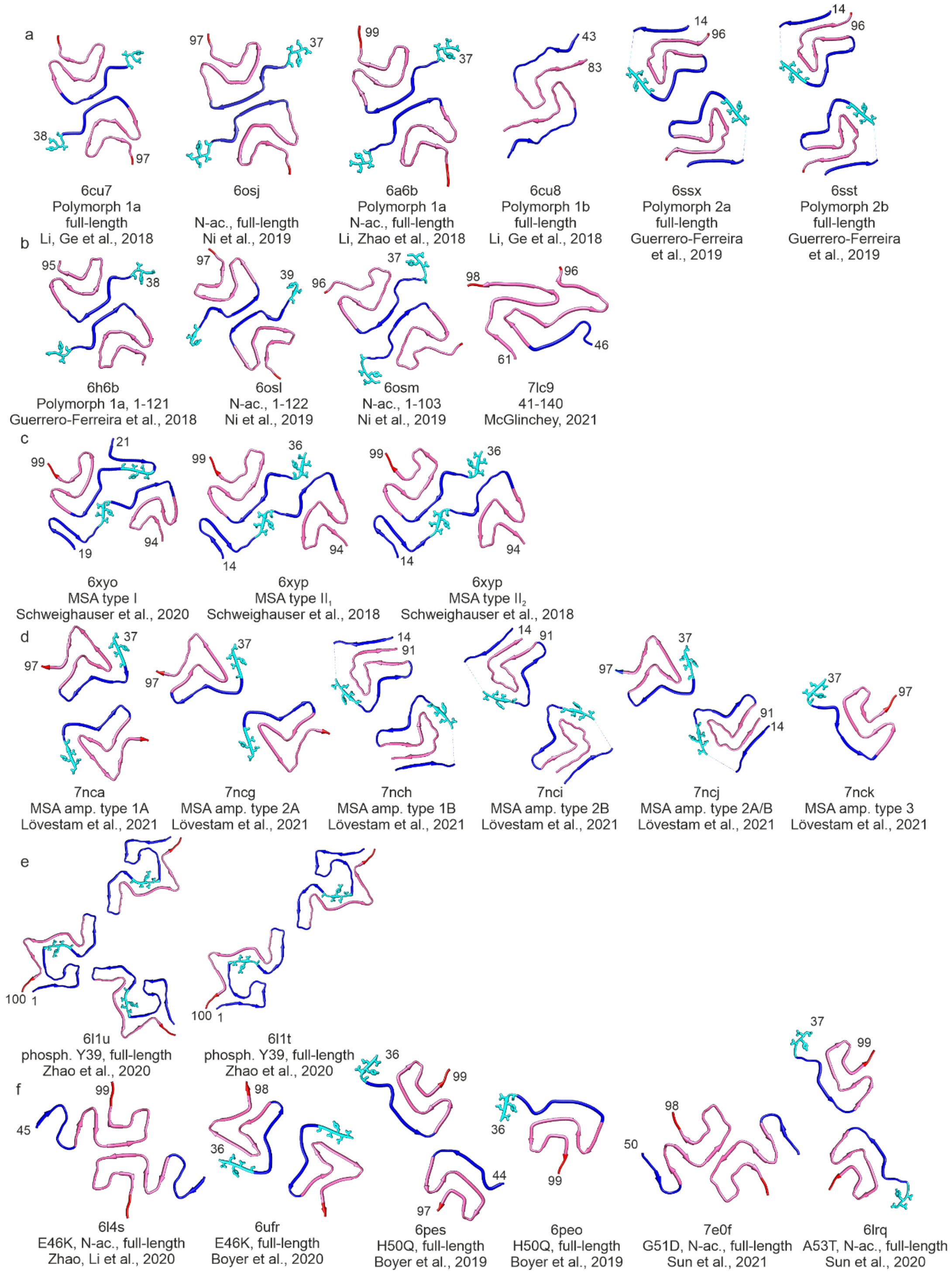

Supplementary Figure 11: Summary of all solved αSyn cryo-EM high resolution structures to date. Fibril structures are illustrated from the top view. Structures of amyloid fibrils (**a**) formed *in vitro* for full length WT αSyn, (**b**) formed in *vitro* using truncated αSyn, (**c**) extracted from MSA patient, (**d**) amplified *in vitro* using MSA fibrils to seed WT αSyn monomers, (**e**) formed *in vitro* from αSyn phosphorylated at Y39 and (**f**) formed *in vitro* from familial PD associated variants. For all structures, the residues in the N-terminal region of the protein visualised in the solved structure is coloured in blue, the P1 region is highlighted in cyan (in ball and stick), the NAC region is pink, and residues observed in the fibril core from the C-terminal region are in red. Note that for some structures the P1 region (as well as other residues in N- and/or C-terminal regions) do not form part of the stable core and are therefore not illustrated here. Below each fibril structure, the PDB file, important characteristics and the associated publication are provided.
