# Supplementary material for: Single residue modulators of amyloid formation in the N-terminal P1-region of α-synuclein
Source: Nat Commun. 2022 Aug 25;13:4986. doi: 10.1038/s41467-022-32687-1 (PMC9411612; doi:10.1038/s41467-022-32687-1)
Supplement: Supplementary file 2 — Reporting Summary [file 41467_2022_32687_MOESM2_ESM.pdf]

nature portfolio

Corresponding author(s): Radford and Brockwell

Last updated by author(s): Jul 27, 2022

Reporting Summary

Nature Portfolio wishes to improve the reproducibility of the work that we publish. This form provides structure for consistency and transparency in reporting. For further information on Nature Portfolio policies, see our Editorial Policies and the Editorial Policy Checklist.

Statistics

For all statistical analyses, confirm that the following items are present in the figure legend, table legend, main text, or Methods section.

n/a	Confirmed	
☐	☒	The exact sample size (*n*) for each experimental group/condition, given as a discrete number and unit of measurement
☐	☒	A statement on whether measurements were taken from distinct samples or whether the same sample was measured repeatedly
☐	☒	The statistical test(s) used AND whether they are one- or two-sided<br>*Only common tests should be described solely by name; describe more complex techniques in the Methods section.*
☒	☐	A description of all covariates tested
☒	☐	A description of any assumptions or corrections, such as tests of normality and adjustment for multiple comparisons
☐	☒	A full description of the statistical parameters including central tendency (e.g. means) or other basic estimates (e.g. regression coefficient) AND variation (e.g. standard deviation) or associated estimates of uncertainty (e.g. confidence intervals)
☐	☒	For null hypothesis testing, the test statistic (e.g. *F*, *t*, *r*) with confidence intervals, effect sizes, degrees of freedom and *P* value noted<br>*Give P values as exact values whenever suitable.*
☒	☐	For Bayesian analysis, information on the choice of priors and Markov chain Monte Carlo settings
☒	☐	For hierarchical and complex designs, identification of the appropriate level for tests and full reporting of outcomes
☒	☐	Estimates of effect sizes (e.g. Cohen's *d*, Pearson's *r*), indicating how they were calculated

Our web collection on statistics for biologists contains articles on many of the points above.

Software and code

Policy information about availability of computer code

Data collection

ThT kinetics were acquired using a plate reader FLUOstar Omega plate reader (BMG Labtech) using the Omega Mars 3.20 RZ (BMG Labtech) software.
SDS PAGE gels were imaged on an Q9 Imager (Uvitec)
TEM data were acquired using a FEI Tecnai T12 electron microscope with the Digital Micrograph software (Gatan).
NMR data were acquired using a Bruker AVANCE III 950MHz spectrometer or Bruker AVANCE III 750MHz spectrometer, data was collected using Topspin 3.2 (Brucker)
Native Mass Spec data were acquired using a Synapt G1 HDMS instrument (Waters Corp., Wilmslow, UK).
CD data were acquired using a ChirascanTM plus CD Spectrometer (Applied Photophysics), using Chirascan version 4.5.1833.0
AFM data were acquired using a Dimension FastScan Bio with FastScan-D-SS probes (Bruker).
Fluorescence imaging was acquired on a a Zeiss LSM880 confocal fluorescent microscope through a 40x 1.0 numerical aperture objective with a 514 nm line for excitation of YFP.
SEC MALS data were collected with a Shimadzu Nexera LC-40 HPLC system (SEC) and Wyatt miniDAWN Treos and Optilab T-rex detectors (MALS)
Western blots were imaged using the ClarityTM ECL Western Sybstrate (Bio-Rad).

Data analysis

ThT data were analysed using Origin Pro (OriginPro 2018b 64Bit) and GraphPad Prism 9.
SDS PAGE band band intensities quantified using Image J 1.52a
NMR data were analysed using NMRpipe for spectra reconstruction and ccpNMR-Analysis software for data analysis
CD was analysed using Microsoft Excel 2013 and Origin Pro (OriginPro 2018b 64Bit).
Native Mass Spec data were analysed using MassLynx 4.1.
For AFM data, heights of single particles were measured automatically using routines written in MATLAB (https://github.com/George-R-Heath/Particle-Detect). Heights and lengths of fibrils were measured either automatically using MATLAB (https://github.com/George-R-Heath/

Correlate-Filaments) or manually in ImageJ for densely packed overlapping fibrils.
Worm body bends were analysed using wrMTrck plugin for ImageJ (available at http://www.phage.dk/plugins/wrmtrck.html).
SEC data were analysed using LabSolutions software (Shimadzu), SEC-MALLS data were analysed using Astra 6.1 (Wyatt).
Western blot densitometry was evaluated using ImageJ 1.52a.
CAMSOL and ZipperDB webservers were used [https://www-cohsoftware.ch.cam.ac.uk/index.php] and [https://services.mbi.ucla.edu/zipperdb/]. Zyggregator is no longer available.

For manuscripts utilizing custom algorithms or software that are central to the research but not yet described in published literature, software must be made available to editors and reviewers. We strongly encourage code deposition in a community repository (e.g. GitHub). See the Nature Portfolio guidelines for submitting code & software for further information.

Data

Policy information about availability of data

All manuscripts must include a data availability statement. This statement should provide the following information, where applicable:

- Accession codes, unique identifiers, or web links for publicly available datasets
- A description of any restrictions on data availability
- For clinical datasets or third party data, please ensure that the statement adheres to our policy

Chemical shift assignments can be accessed using BMRB accession numbers BMRB 51120 [https://dx.doi.org/10.13018/BMR51120](WT-αSyn, pH 7.5) and BMRB 51121 [https://dx.doi.org/10.13018/BMR51121] (ΔP1 αSyn, pH 7.5). Source data are available online in the University of Leeds data repository (https://doi.org/10.5518/1051).

Field-specific reporting

Please select the one below that is the best fit for your research. If you are not sure, read the appropriate sections before making your selection.

[x] Life sciences   [ ] Behavioural & social sciences   [ ] Ecological, evolutionary & environmental sciences

For a reference copy of the document with all sections, see nature.com/documents/nr-reporting-summary-flat.pdf

Life sciences study design

All studies must disclose on these points even when the disclosure is negative.

Sample size	No statistical methods were used to determine sample size. Experiments were repeated to ensure reproducibility.
Data exclusions	No data were excluded from the study
Replication	At least 3 replicates were performed for each ThT experiment with two biological replicates, reproducibility between experiments validated the experimental approach. Sample sizes in Caenorhabditis elegans experiences were n=10. For Mass Spec experiments n=2. For NMR and CD and SEC-MALLS experiments n=1,and for Western blot analysis n=3. All experiments showed good reproducibility.
Randomization	This is not relevant for our study, because no grouping was needed.
Blinding	Investigators were not blinded to group allocation as no grouping was needed for this study.

Reporting for specific materials, systems and methods

We require information from authors about some types of materials, experimental systems and methods used in many studies. Here, indicate whether each material, system or method listed is relevant to your study. If you are not sure if a list item applies to your research, read the appropriate section before selecting a response.

Materials & experimental systems

n/a	Involved in the study	
[ ]	[x]	Antibodies
[x]	[ ]	Eukaryotic cell lines
[x]	[ ]	Palaeontology and archaeology
[ ]	[x]	Animals and other organisms
[x]	[ ]	Human research participants
[x]	[ ]	Clinical data
[x]	[ ]	Dual use research of concern

Methods

n/a	Involved in the study	
[x]	[ ]	ChIP-seq
[x]	[ ]	Flow cytometry
[x]	[ ]	MRI-based neuroimaging

Antibodies

Antibodies used	Proteins were visualised using a mouse anti-GFP antibody (anti-GFP (1:1,000) (BioLegend clone B34, 902601)) or mouse anti-tubulin antibody (1:5,000) (Sigma clone DM1A monoclonal, T9026), followed by an anti-mouse horseradish peroxidase-coupled secondary antibody (1:5,000) (Cell Signaling Technology, 7076S).
Validation	All antibodies are commercially available and were tested by the manufacturer and are routinely used in the literature.

Animals and other organisms

Policy information about studies involving animals; ARRIVE guidelines recommended for reporting animal research

Laboratory animals	This study used Caenorhabditis elegans (strains obtained from CGC (University of Minnesota). Maintenance and genetic manipulation of animals was done using well established protocols. Day 0, 5 and 10 worms were analysed in this study.
Wild animals	No wild animals were used.
Field-collected samples	No field collected samples were used
Ethics oversight	No ethical approval required for Caenorhabditis elegans.

Note that full information on the approval of the study protocol must also be provided in the manuscript.
