# Supplementary material for: Single residue modulators of amyloid formation in the N-terminal P1-region of α-synuclein
Source: Nat Commun. 2022 Aug 25;13:4986. doi: 10.1038/s41467-022-32687-1 (PMC9411612; doi:10.1038/s41467-022-32687-1)
Supplement: Supplementary file 3 — Source Data [file 41467_2022_32687_MOESM3_ESM.zip › SourceData_Western_Blots_Uncroppped.docx]

Western blot analysis – uncut gels
